# Alzheimer’s Disease: From Molecular Mechanisms to Promising Therapeutic Strategies

**DOI:** 10.3390/ijms26199444

**Published:** 2025-09-26

**Authors:** Anna V. Ivanova, Alexandra D. Kutuzova, Ilia A. Kuzmichev, Maxim A. Abakumov

**Affiliations:** Department of Medical Nanobiotechnology, N.I. Pirogov Russian National Research Medical University, 117997 Moscow, Russia

**Keywords:** Alzheimer’s disease, pathogenesis, therapeutic targets, PET imaging, radiopharmaceuticals

## Abstract

Alzheimer’s disease (AD) is the most common cause of dementia worldwide, and there are still no strategies to slow or prevent its clinical progression. Significant financial and research resources have been invested into studying the pathology of AD. However, its pathogenesis is not fully understood. This review provides a comprehensive analysis of current understanding of AD pathogenesis, including classical hypotheses (amyloid cascade, tau pathology, neuroinflammation, oxidative stress), emerging mechanisms (cellular senescence, endoplasmic reticulum stress, ubiquitin-proteasome system dysfunction), and alternative mechanisms (cholinergic dysfunction, glutamate excitotoxicity, disruption of the microbiota–gut–brain axis, and autophagy). Schematic illustrations summarize the relationships between the hypotheses and their role in the pathogenesis of AD. Particular attention is paid to the systematization of promising biological targets and the analysis of modern ligands of various nature, including small molecules, peptides, antibodies and their fragments, natural compounds, as well as innovative hybrid and multifunctional structures. A separate section is devoted to radiopharmaceuticals for PET imaging (Florbetaben, Flortaucipir, etc.) and promising therapeutic agents. Thus, in this review we (1) systematize modern concepts of AD pathogenesis, including classical, emerging mechanisms and alternative hypotheses; (2) conduct a comparative analysis of ligand classes (small molecules, peptides, antibodies, etc.) and their therapeutic potential; and (3) discuss the clinical prospects of radiopharmaceuticals for PET imaging and targeted therapy. The work provides a comprehensive analysis of modern approaches, which can help in the development of more effective drugs against AD.

## 1. Introduction

The history of Alzheimer’s disease (AD) began with its description in 1906 by the German neurologist Alois Alzheimer, who identified its main features—amnesia, changes in thinking and behavioral disorders. According to the prevailing amyloid cascade hypothesis, AD pathogenesis is driven by the abnormal accumulation in the human brain of beta-amyloid (Aβ) and tau protein, which form extracellular senile plaques and intracellular neurofibrillary tangles (NFTs), respectively [1]. The amyloid cascade hypothesis, which posits Aβ deposition as the initiating event, has long dominated the field and underpinned most drug development efforts. As mentioned earlier, the first characteristic morphological sign of AD is the formation of senile plaques, which consist of an insoluble aggregated peptide called Aβ. This peptide is 39–43 residues long and is derived from the Aβ precursor protein (APP) [2], found in many tissues, including neuronal synapses. APP is involved in neuroplasticity, synapse formation, and is necessary for the survival of nerve cells. Notably, Aβ, being a product of proteolytic cleavage of APP, has pronounced fibrillogenic properties and its oligomers are toxic to nerve cells, causing their degeneration and death [3,4]. The second characteristic morphological feature of AD is the disruption of the cytoskeleton of nerve cells and the accumulation of NFTs inside them, consisting of a hyperphosphorylated form of a microtubule-associated protein called tau protein [5]. According to the prevailing model, amyloid fibrils are formed first during the neurodegenerative process, disrupting neuronal function, followed by the formation of NFTs. Together, these two processes lead to the degeneration and death of neurons, primarily in the cortex and hippocampus, ultimately causing progressive cognitive decline [6,7]. Thus, the interplay of Aβ and tau pathology is a cornerstone of AD pathogenesis.

However, the etiology of AD is complex and varied, and the exact mechanisms underlying its occurrence are still not fully understood. In addition to the key role of Aβ and tau protein, other factors such as acetylcholine deficiency, neuroinflammation, oxidative stress, bioelement imbalance, glutamate excess, insulin resistance, intestinal microbiome disturbances, cholesterol homeostasis disturbances, mitochondrial dysfunction, autophagy disturbances, endoplasmic reticulum stress, and cellular senescence are also implicated in the pathogenesis of AD [2,8,9,10,11]. This expansive list of pathological factors presents a significant challenge for therapy but also reveals a vast array of potential diagnostic and therapeutic targets. It should be noted that these factors also serve as the basis for clinical diagnosis and treatment strategies. Hypotheses related to these pathogenic factors serve as potential targets for drug development. Yet, despite more than a century passing since the first description of AD in 1906 [12], and significant progress made in understanding its pathogenesis, improving diagnosis and treatment [13,14] existing therapeutic approaches remain insufficiently effective for correcting cognitive impairment and do not meet the urgent need for effective treatment. This review aims to address this critical challenge by providing a unique perspective. We move beyond a conventional summary of therapeutic strategies to focus on the pivotal role of radiopharmaceuticals in both diagnosing AD and enabling the development of novel therapies. To this end, our review systematizes (1) key biological targets of AD, (2) vector molecules with proven tropism to these targets, and (3) promising therapeutic radiopharmaceuticals and a comparative analysis of existing analogues with an assessment of their advantages and clinical limitations. While numerous reviews have detailed therapeutic strategies for AD, this work aims to provide a unique integration of these approaches with the rapidly evolving field of radiopharmaceuticals. By integrating the discussion of pathogenic mechanisms with the advanced tools designed to detect and treat them, we highlight how diagnostic tracers are paving the way for novel theranostic applications and resolving critical bottlenecks in drug development.

## 2. Mechanisms of AD Development

**Figure 1:**
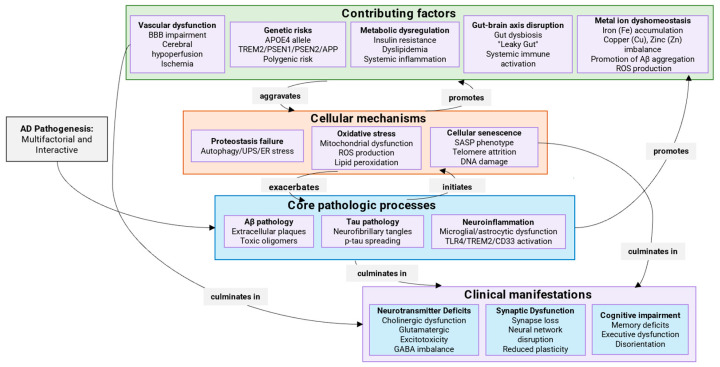
Integrated multifactorial model of AD Pathogenesis: interplay between core pathologies, modifiable risk factors and clinical outcomes. This model illustrates how genetic, vascular, metabolic, and environmental risk factors (e.g., APOE ε4, BBB impairment, gut dysbiosis, metal imbalance) converge to disrupt cellular homeostasis, promoting Aβ/tau pathology through oxidative stress, proteostasis failure, and neuroinflammation. These processes drive synaptic loss, neurotransmitter deficits, and neural network dysfunction, ultimately leading to cognitive decline. The diagram emphasizes the interconnectedness of diverse mechanisms in AD progression.

Many hypotheses have been put forward to explain the pathogenesis of AD, but a unified theory has not yet been found, probably due to the complex nature of AD. There are two main forms of AD: sporadic (SAD) (95% of cases) and familial (FAD) (1–5% of cases) [15,16]. SAD is the most common type of the disease, known as late-onset AD, typically appears after age 65 and is influenced by a combination of genetic and non-genetic factors such as the environment and various comorbidities [17,18,19]. In contrast to the Mendelian inheritance of FAD, the genetic architecture of SAD is complex and polygenic. The most significant genetic risk factor is the apolipoprotein E ε4 allele (APOE4), which can increase the risk of developing AD by 3–15 times depending on the dosage and is associated with an earlier age of onset [20]. The APOE4 genotype is thought to influence multiple pathological processes, including impaired Aβ clearance, increased Aβ aggregation, dysregulated lipid metabolism, and exacerbated neuroinflammation. Beyond APOE, genome-wide association studies (GWASs) have identified over 80 additional genetic loci that contribute to polygenic risk for SAD [21,22]. Many of these genes are highly expressed in microglia and are involved in innate immune responses (e.g., TREM2, CD33, INPP5D), underscoring the critical role of neuroinflammation in SAD pathogenesis. Other implicated loci are involved in endocytosis (BIN1, PICALM), lipid metabolism (ABCA7), and synaptic function [23,24]. The cumulative effect of these common variants, each conferring a small amount of risk, combines to significantly influence an individual’s susceptibility. Crucially, this genetic predisposition interacts with a multitude of non-genetic factors. Vascular contributions, including hypertension, atherosclerosis, and cerebral small vessel disease, are major drivers of pathology in SAD, often leading to mixed dementia pathologies [25]. Vascular dysfunction can impair cerebral blood flow, reduce the clearance of Aβ and other toxins across the blood–brain barrier, and promote hypoperfusion and ischemia, thereby accelerating neurodegenerative processes [26]. Furthermore, modifiable lifestyle and environmental factors play a substantial role in modulating risk. Factors such as mid-life obesity, type 2 diabetes, physical inactivity, low educational attainment, depression, and hearing loss have been identified as significant contributors. The mechanisms through which these factors act is diverse, including promoting systemic inflammation, oxidative stress, and reducing cognitive reserve. This highlights that a considerable portion of SAD risk is theoretically preventable through targeted public health interventions and lifestyle modifications across the lifespan [27]. Each of the genetic factors influences one or more known pathogenetic mechanisms: increased production and aggregation of Aβ; decreased clearance and degradation of Aβ; increased inflammation and resistance to γ-secretase activity, which thus leads to neurodegeneration. Non-genetic factors may increase the risk of developing AD, comorbidities and complications. They are capable of influencing biological processes and changing genetic predisposition, thereby contributing to the onset or progression of the disease. This complex network of interacting genetic and non-genetic factors leading to the pathogenesis of AD is clearly illustrated in (Figure 1). Particular attention should be paid to the fact that in old age, concomitant diseases such as cerebrovascular pathologies and hippocampal sclerosis are often observed, which complicates the identification of a direct set of symptoms and manifestations of AD [28,29,30]. FAD is predominantly characterized by autosomal dominant genetic mutations in the amyloid precursor protein (APP), presenilin 1 (PS1), and presenilin 2 (PS2) genes, and is typically characterized by an early onset, usually in middle age, around 45 years [15]. Alterations in the PS1 and PS2 genes result in increased production of Aβ_42_ in cells, which in turn accelerates amyloid deposition in the brain. SAD and FAD are comparable in most clinical aspects, including the rate of disease progression and biomarker profiles. In most clinical aspects, SAD and FAD forms of AD are comparable, including the rate of disease progression and biomarker profiles. Developing a comprehensive theoretical framework linking the genetic basis, molecular mechanisms and clinical phenotypes of AD is extremely challenging. Current limitations in AD research significantly impede a comprehensive understanding of its pathophysiology, which is likely due to the simultaneous existence of several competing theories (these will be discussed in detail in the following sections).

### 2.1. Cholinergic Hypothesis

**Figure 2:**
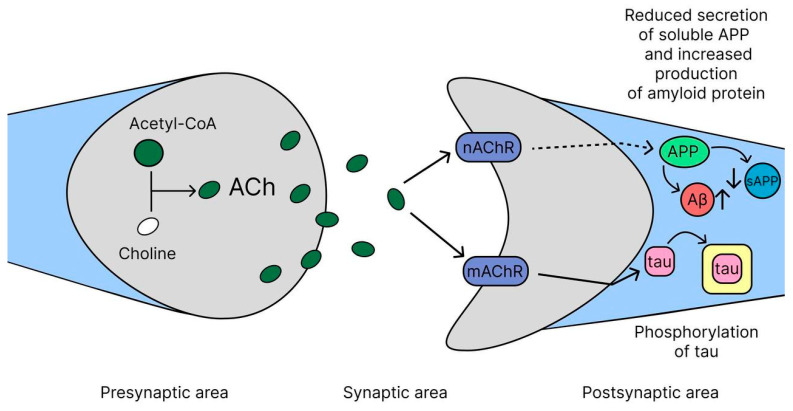
Cholinergic hypothesis of AD: key mechanisms of neurodegeneration. The figure summarizes key mechanisms linking cholinergic dysfunction to AD neurodegeneration. Solid arrows indicate direct metabolic conversions or interactions, while dashed arrows represent indirect or regulatory influences. Reduced Ach synthesis from choline and acetyl-CoA leads to presynaptic deficits and impaired signaling through nicotinic (nAChR) and muscarinic (mAChR) receptors. This disrupts synaptic transmission and promotes amyloidogenic processing of APP, increasing Aβ production while decreasing neuroprotective sAPPα. Concomitant tau hyperphosphorylation further exacerbates synaptic failure and neuronal damage, creating a vicious cycle that drives cognitive decline.

The cholinergic hypothesis represents one of the earliest and most clinically successful frameworks for understanding cognitive decline in AD, leading to the first approved pharmacological therapies [31]. According to this hypothesis, the main cause of the disease symptoms is damage to cholinergic neurons in the nucleus basalis of Meynert (NBM), which play a key role in the production of acetylcholine. This leads to a decrease in choline acetyltransferase (ChAT) activity in the cerebral cortex and hippocampus, which causes a deficiency of acetylcholine (ACh), an important neurotransmitter involved in learning, memory, and other cognitive functions (Figure 2). Cholinergic neurons in the basal forebrain, predominantly located in the medial septal nucleus (MSN), diagonal band of Broca (DBB), NBM, and substantia innominate (SI), are essential components of the central cholinergic system, making significant contributions to the regulation of cognitive functions, attention, and memory. However, in AD they become damaged and, as a consequence, degeneration and loss of neurons occurs in AD due to the disruption of nerve growth factors (NGF) [32,33,34,35]. Ach is synthesized in the cytoplasm of cholinergic neurons, mainly in their nerve endings, from choline and acetyl-CoA under the action of the enzyme choline acetyltransferase (ChAT). After synthesis, ACh is transported into synaptic vesicles (special bubbles) via the vesicular acetylcholine transporter (VAChT), where it is stored until release. When the nerve impulse reaches the terminal of the neuron, the membrane is depolarized, causing calcium channels to open and ACh to be released into the synaptic cleft. After performing its function, ACh is broken down in the synaptic cleft by the enzyme acetylcholinesterase (AChE) into choline and acetic acid. Choline is then taken back into the neuron via choline transporters and used to resynthesize ACh [9,36,37]. Thus, ACh synthesis is a complex but well-organized process that ensures the transmission of nerve impulses and the maintenance of cognitive functions, and its disruption in AD leads to a deterioration in its physiological functions associated with learning, memory, movement regulation and the sleep cycle [38,39]. The idea of the key role of acetylcholine deficiency in the development of AD formed the basis for the creation of the first drugs for its treatment—AChE inhibitors: donepezil, rivastigmine and galantamine. These drugs, approved more than 20 years ago, still remain the mainstay of AD therapy in clinical practice [40]. These drugs slow the breakdown of Ach, increasing its levels in the brain and temporarily improving cognitive function. Although this concept does not fully explain the complex pathology of AD, which has led to other theories such as the amyloid hypothesis and the tau hypothesis, the cholinergic hypothesis continues to play an important role in understanding the pathophysiology of the disease.

Thus, while the cholinergic hypothesis primarily addresses the symptomatic manifestations of AD rather than its root cause, it established a critical foundation for AD drug discovery. The modest but reliable efficacy of AChE inhibitors confirmed that targeting neurotransmitter systems could alleviate cognitive symptoms, setting the stage for the development of future therapies aimed at the underlying Aβ and tau pathologies discussed in the following sections.

### 2.2. Amyloid Hypothesis

**Figure 3:**
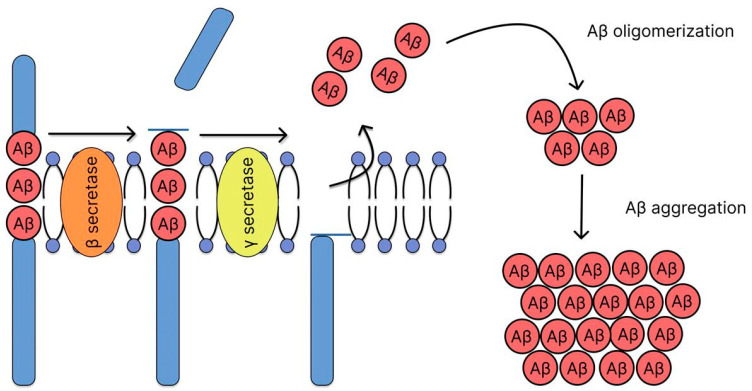
Amyloidogenic pathway of APP processing and Aβ formation. The amyloidogenic processing of APP involves sequential cleavage by β-secretase (BACE1) and γ-secretase, generating neurotoxic Aβ peptides (e.g., Aβ_42_). These peptides oligomerize and aggregate into insoluble plaques, disrupting synaptic function, triggering neuroinflammation, and promoting tau pathology—key events driving neurodegeneration in AD.

The amyloid hypothesis has been the dominant theory for decades, positing that the accumulation of Aβ plaques plays a key role in AD pathogenesis. Aβ is formed by the cleavage of the APP by a proteolytic mechanism that occurs via two different pathways: amyloidogenic and non-amyloidogenic. In the non-amyloidogenic pathway, APP molecules are cleaved at the α-secretase site within the Aβ domain, resulting in the release of the soluble exodomain (sAPPα) and the C-terminal fragment (CTF83). The remaining transmembrane fragment is then cleaved by γ-secretase within the transmembrane domain, releasing the non-amyloidogenic peptide P3 (Aβ_17–40_ or Aβ_17–42_) and the APP transcriptional regulator intracellular domain (AICD). The amyloidogenic (Figure 3) pathway involves the sequential cleavage of APP by the aspartate proteinase β-secretase (also known as β-APP cleavage enzyme or BACE-1), which releases the soluble exodomain (sAPPβ) and the C-terminal fragment of CTF99. This fragment, in turn, is cleaved by another aspartate proteinase, γ-secretase, which leads to the formation of the intracellular domain of the APP transcriptional regulator (AICD) and the release of the 39–42 amino acid long amyloid Aβ. The length of the peptide depends on the site of γ-secretase cleavage, which creates variability in the hydrophobic C-termini connected to the hydrophilic N-terminal domain [41]. In 2014, the three-dimensional structure of human γ-secretase was determined for the first time using cryo-electron microscopy [42]. This enzyme consists of 19 transmembrane segments that form a horseshoe-shaped structure and a large extracellular domain (ECD) of the nicastrin subunit located above the cavity formed by the transmembrane segments. This structure has become key to understanding the mechanisms of γ-secretase. γ-secretase is a complex of four proteins: PS1 and PS2, nicastrin, presenilin enhancer 2 (PEN-2), and anterior pharyngeal defective protein 1 (APH-1). Presenilin, the catalytic subunit, is activated by self-processing, resulting in the formation of N- and C-terminal fragments containing aspartate protease sites required for enzymatic activity. The remaining components of the complex (nicastrin, presenilin enhancer 2, and anterior pharyngeal defective protein 1) regulate γ-secretase activity in response to physiological signals [41,43]. Cleavage of APP by γ-secretase results in the formation of Aβ, making this enzyme an important target for the development of AD therapies [44]. The two major peptides resulting from proteolytic cleavage are 40 (Aβ_40_) and 42 (Aβ_42_) amino acid residues long. Aβ_42_ exhibits a greater tendency to aggregate in vivo and is often considered more toxic [45,46]. The mechanism of toxicity of Aβ aggregates is not fully understood, but there are several hypotheses [17,47]. According to one of them, Aβ may contribute to the development of pathology in AD due to the loss of its physiological functions during the aggregation process [48]. Due to their high tendency to aggregation, Aβ monomers form highly organized oligomeric structures, protofibrils and amyloid fibrils. These aggregates cause a chain of pathological disturbances, including synaptic dysfunction, microglial activation followed by inflammation and massive neuronal death [2,49,50]. The amyloid hypothesis has provided key insights into the mechanisms of AD. Based on this, drugs have been developed to slow the disease. However, the clinical translation of the amyloid hypothesis has proven immensely challenging, revealing significant limitations and necessitating a critical reassessment of its role in therapeutic development. Currently, antibodies such as aducanumab, lecanemab, and donanemab are proving effective in proving the key role of Aβ in AD, but their clinical utility remains questionable and requires further confirmation [17,18]. Despite these clear biomarker effects, their clinical utility remains questionable and highly controversial, with debates centering on the modest cognitive benefits observed and their risk-benefit profile. The accelerated approvals of aducanumab and lecanemab, alongside the history of numerous failed anti-amyloid trials (e.g., with gamma-secretase inhibitors and BACE inhibitors), highlight a persistent disconnect between amyloid removal and substantial cognitive improvement in patients with symptomatic AD. A discussion on why targeting amyloid may have limited clinical success despite clear biomarker changes is crucial. Several key explanations have emerged for this discrepancy: (1) The timing of intervention may be too late; by the clinical stage of AD, Aβ accumulation has already triggered widespread downstream pathologies (e.g., tau tangles, neurodegeneration) that become self-sustaining and are not halted by amyloid removal. (2) Soluble Aβ oligomers, rather than insoluble plaques, may be the primary toxic species responsible for synaptic dysfunction, and not all anti-amyloid therapies effectively target these oligomers. (3) The significant adverse effects, such as Amyloid-Related Imaging Abnormalities (ARIAs), associated with potent amyloid-lowering antibodies, limit their tolerable dosing and thus their potential efficacy. It should be noted that the amyloid cascade hypothesis remains controversial. The theory faces difficulties in explaining the weak correlation between amyloid load and cognitive decline in later stages and the existence of non-amyloid pathways in the disease etiology. Given the growing recognition of the critical role of tau protein and other mechanisms, it is now widely accepted that Aβ pathology is a key initiating trigger, but not the sole driver, of AD pathogenesis. Therefore, while amyloid remains a primary biomarker for diagnosis and a validated target for drug development, its targeting as a monotherapy in symptomatic stages appears to have limited clinical success. Future strategies may require combination therapies targeting both amyloid and downstream processes like tau pathology or neuroinflammation, or a much earlier intervention in the preclinical disease stage. Further study of the pathological sequence and interaction between Aβ and tau protein appears to be of utmost importance for understanding the mechanisms of disease development [51,52]. This understanding of Aβ role as an initial trigger, rather than the sole executor of neuronal death, naturally shifts the focus to the downstream mechanisms it activates, most notably the pathological processing of tau protein.

### 2.3. Tau Hypothesis

**Figure 4:**
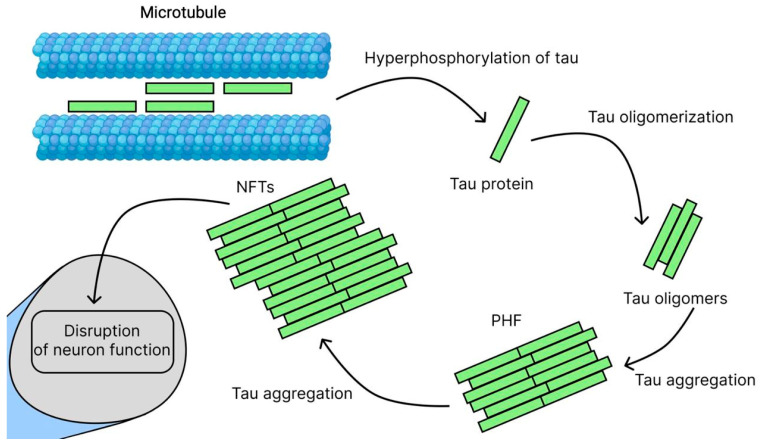
Molecular mechanisms of tau protein hyperphosphorylation and NFT formation. Hyperphosphorylation of tau protein leads to its detachment from microtubules, causing cytoskeletal instability and disruption of neuronal function. Misfolded tau forms soluble oligomers, which further aggregate into PHFs and NFTs. This progression from dysfunctional tau to NFT deposition drives synaptic impairment, disrupted cellular homeostasis, and ultimately neurodegeneration in AD.

Building on the amyloid cascade framework, the tau hypothesis addresses the critical downstream pathology that more directly correlates with neurodegeneration and cognitive decline. One of the key manifestations of AD is damage to nerve cells, within which tightly packed filaments form, but unlike amyloid plaques, they accumulate intracellularly, forming NFTs [5]. These pathological formations consist of insoluble protein threads formed from microtubule-associated protein—tau protein [53]. The functional activity of tau protein is under strict control of the phosphorylation system, implemented by a complex of protein kinases and phosphatases [54]. This process is mediated by the activation of the main regulatory kinases—GSK3β [55] and CDK5 [56]—which leads to their synergistic effect on the phosphorylation process [57]. In pathology, this balance is disrupted, leading to hyperphosphorylation (Figure 4). This cascade of molecular events in the cell body or dendrites leads to dissociation of tau protein from microtubules, conformational changes, and accumulation of pathological aggregates (oligomers, paired helical filaments PHF, and NFT), ultimately causing disruption of neuronal functions with subsequent cell death [58]. It is important to note that in addition to phosphorylation, other post-translational modifications (truncation, glycosylation, glycation, and sumoylation) also contribute to protein aggregation and increased toxicity [59,60,61,62]. Of particular danger are tau oligomers, which have the ability to spread between neurons through synaptic contacts, triggering a cascade of neurodegenerative changes. The clinical significance of these processes is confirmed by a pronounced correlation between the degree of accumulation of pathological tau protein and the severity of cognitive impairment [63]. Current research is focused on the relationship between tau protein deposition, brain atrophy and glucose metabolism disorders. Despite the modest success of the first clinical trials, new therapeutic approaches are actively being developed, including kinase inhibitors, anti-aggregation compounds, immunotherapy methods, autophagy-stimulating drugs, and protein targeted degradation technologies (PROTACs) [64,65]. These promising directions open up new possibilities for the development of effective treatments for AD.

### 2.4. Neuroinflammation Hypothesis

**Figure 5:**
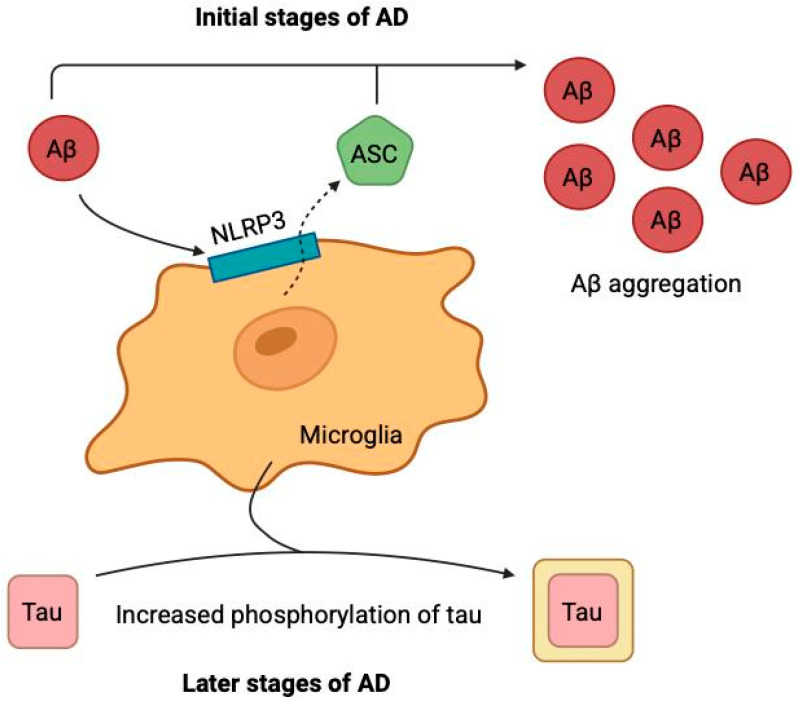
The role of microglia in the pathogenesis of AD: interaction with Aβ and tau protein, activation of neuroinflammation. The figure illustrates the dual role of microglia in AD progression. Solid arrows indicate direct interactions or processes, while dashed arrows represent indirect or subsequent pathological effects. Microglia play a critical yet dual role in AD pathogenesis. Initially, they attempt to clear Aβ aggregates via phagocytosis, but chronic exposure to Aβ activates them through inflammasomes (e.g., NLRP3), triggering pro-inflammatory cytokine release. This response enhances Aβ aggregation and tau hyperphosphorylation, forming a vicious cycle: neuroinflammation promotes tau pathology and neuronal damage, while accumulated Aβ and tau further sustain microglial activation. Ultimately, chronic inflammation accelerates neurodegeneration, linking innate immunity to core AD mechanisms.

While the amyloid and tau hypotheses focus on proteinopathies, the neuroinflammation hypothesis highlights the crucial role of the brain’s innate immune response, which can both respond to and exacerbate these core pathologies. Alois Alzheimer was the first to recognize the involvement of microglia in the development of the disease that was later named after him [66]. It was only in 1990 that it was first demonstrated that microglia interact with both Aβ and tau protein [67,68] triggering a cascade of pathological reactions. Biochemical studies have revealed that Aβ binds to microglial membrane receptors (TREM2, TLR4, CD33), activating proinflammatory signaling pathways, including the NLRP3 inflammasome with subsequent formation of ASC particles [69], and the p38 MAPK cascade [70], which leads to chronic neuroinflammation. In the early stages, this process is protective: microglia efficiently phagocytose Aβ via a TREM2-dependent mechanism. However, mutations in the receptor expressed on myeloid cells 2 (TREM2) or prolonged exposure to Aβ lead to microglial dysfunction, manifested by decreased amyloid clearance, increased oxidative stress and neurotoxicity. As a result, a pathological cycle is formed (Figure 5): accumulation of Aβ → activation of NLRP3/ASC → chronic neuroinflammation → neuronal damage → progression of tau pathology, where extracellular ASC particles further enhance the aggregation of Aβ and tau protein [69,71]. Importantly, tau protein, unlike Aβ, preferentially interacts with microglia after exiting damaged neurons, enhancing and prolonging the inflammatory response in the late stages of the disease. Therapeutic strategies aimed at correcting microglial receptor activity and suppressing neuroinflammation represent a promising direction in the treatment of the disease. Numerous drugs that target inflammation-related receptors, signaling pathways, and proinflammatory cytokines are currently in clinical trials [69]. Thus, neuroinflammation is no longer seen as a passive response but as an active driver of AD pathogenesis, creating a vicious cycle with Aβ and tau that amplifies neuronal damage and creates a self-sustaining pathological environment. This chronic inflammatory state also contributes to other downstream processes, such as oxidative stress and cellular dysfunction.

### 2.5. Oxidative Stress Hypothesis

**Figure 6:**
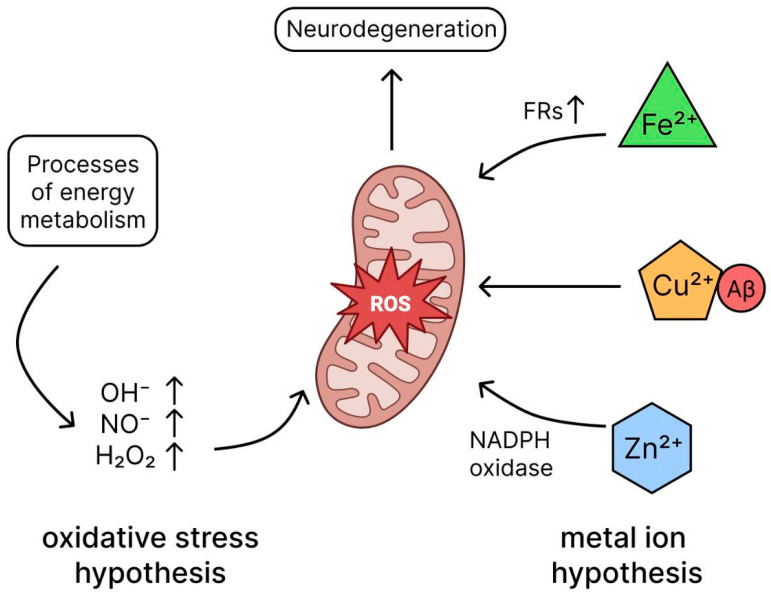
Combined effects of oxidative stress and metal imbalance on neurodegeneration in AD. This figure illustrates the synergistic pathological cycle between oxidative stress and metal ion dyshomeostasis, two core mechanisms in AD. The oxidative stress hypothesis (left) highlights how impaired energy metabolism and mitochondrial dysfunction lead to excessive production of ROS, resulting in oxidative damage to lipids, proteins, and DNA. Concurrently, the metal ion hypothesis (right) emphasizes the disruption of metal homeostasis (e.g., iron, copper, zinc), which further amplifies oxidative stress through Fenton chemistry and promotes Aβ aggregation and tau hyperphosphorylation.

Accumulating evidence firmly establishes that the brain in AD is under increased oxidative stress, which plays a critical role in the pathogenesis of neuronal degeneration and death [72,73]. The main cause of oxidative stress is considered to be an imbalance between the production of free radicals (FRs) and the activity of the body’s antioxidant systems [74,75]. The main sources of reactive oxygen species (ROS) in cells are energy exchange processes, in particular the reaction of electron transfer from donor to acceptor. These reactions generate highly reactive intermediates such as OH·, OH^−^, NO· and H_2_O_2_ [76], which can damage cellular structures. Mitochondria, which are the main source of ATP and simultaneously a significant supplier of ROS, play a special role in the generation of FRs (Figure 6) [77]. When mitochondrial function is impaired, electrons leak from the respiratory chain, which increases oxidative stress and, according to modern concepts, contributes to the development of AD. Thus, mitochondrial dysfunction and associated oxidative stress represent an important link in the pathogenesis of AD. The brain is particularly vulnerable to oxidative stress due to a number of key factors. Firstly, it contains high levels of iron, which can catalyze the formation of free radicals. Secondly, the abundance of polyunsaturated fatty acids in neuronal membranes makes them a target for lipid peroxidation. In addition, the brain consumes a huge amount of oxygen (about 20% of the total volume) and has a high metabolic turnover, which inevitably leads to the formation of reactive oxygen species (ROS) during energy metabolism. In AD, this process is exacerbated by the accumulation of Aβ oligomers, which themselves stimulate the generation of ROS, creating a vicious cycle of oxidative damage [78]. Mitochondrial dysfunction plays a critical role in this: due to a decrease in ATP synthesis and electron leakage in the respiratory chain, DNC, proteins and lipids are damaged, which further inhibits energy metabolism and leads to neuronal death [79]. Normally, brain cells are protected by antioxidant systems, but in patients with AD this protection is weakened [80]. Oxidative stress, in the early stages of AD, is considered as an integral link connecting the main pathogenetic mechanisms of AD—amyloidogenesis, tau pathology and neuroinflammation. This opens up prospects for the development of complex therapeutic strategies aimed at correcting the redox balance in combination with an impact on other links in pathogenesis [81]. Given these links between oxidative stress and other mechanisms of AD development, antioxidants have emerged as promising treatments for AD, with positive results seen in animal models [82]. Clinical data on the effectiveness of antioxidants in AD are contradictory, most clinical trials (vitamin E [83], ginkgo biloba [84], curcumin [85], etc.) have not shown significant improvement, but research is ongoing. Future efforts should be directed towards optimizing drug dosage and initiating antioxidant therapy early in the disease course to potentially improve outcomes [85], and the interaction between Aβ and oxidative stress and their sequence in AD requires further investigation [86]. Therefore, oxidative stress acts as a common downstream pathway through which diverse initial insults—Aβ accumulation, tau pathology, and neuroinflammation—converge to execute neuronal damage. This central role makes the redox system an attractive, albeit challenging, therapeutic target for breaking the vicious cycle of AD pathogenesis. A critical factor contributing to this heightened oxidative stress in the AD brain is the dyshomeostasis of biologically active metal ions, particularly iron, copper, and zinc.

### 2.6. Metal Ion Hypothesis

Under physiological conditions, trace elements maintain homeostasis of the neuronal microenvironment, which consists of metal ions. However, in AD this balance is disturbed, which is convincingly confirmed by postmortem analyses of amyloid plaques, revealing excessive accumulation of copper (×5.7), iron (×2.8) and zinc (×3.1) compared to the norm [87]. The obtained results not only confirm the association of Fe^2+^/Cu^2+^/Zn^2+^ imbalance with the development of AD, but also reveal the mechanism of low efficacy of antiamyloid drugs due to the formation of metal-Aβ aggregates resistant to degradation. Thus, the development of drugs to correct metal balance can simultaneously reduce the formation of amyloid plaques and reduce oxidative stress [88]. Copper complexes with Aβ in the presence of endogenous reductants catalyze the formation of ROS from molecular oxygen, whereas iron-dependent oxidative stress causes the release of Fe^2+^ from iron-containing proteins, leading to the Fenton reaction, ferroptosis, and lipid peroxidation (Figure 6). Both mechanisms contributing to neuronal death [89,90,91]. Zinc modulates neuronal signaling via NMDA receptors, preventing glutamate-induced excitotoxicity. Experimental data indicate that Zn^2+^ ions competitively inhibit Ca^2+^ binding to NMDA receptors, reducing pathological hyperexcitability of neurons [92]. Zinc supplementation stimulates the conversion of pro-BDNF to mature BDNF, as confirmed in vivo in AD models (40% increase in synaptophysin) [93]. Thus, zinc deficiency is critical in the context of glutamate excitotoxicity and synaptic dysfunction in AD. Metal imbalance plays a key role in the pathogenesis of AD, promoting amyloidosis, tauopathy, oxidative stress and neurodegeneration, making metal chelators a promising therapeutic approach to reduce redox stress and restore ion homeostasis [94,95,96]; however, to realize their clinical potential, problems associated with side effects and low blood–brain barrier (BBB) permeability need to be overcome [97]. Thus, metal ion dyshomeostasis acts as a key upstream event that exacerbates oxidative stress and directly catalyzes protein misfolding, thereby integrating multiple pathological pathways in AD.

### 2.7. Glutamate Excitotoxicity

**Figure 7:**
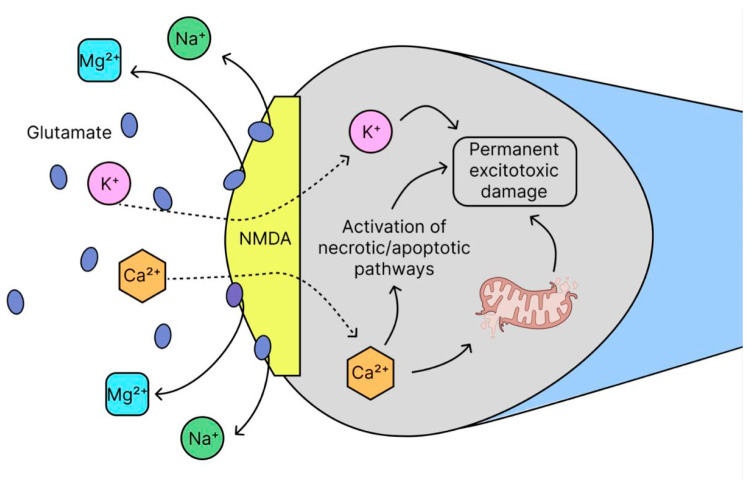
Mechanism of glutamate excitotoxicity via NMDA receptors in AD. The diagram compares NMDA receptor function under physiological and AD conditions. Solid arrows represent ion fluxes and direct activation, while dashed arrows indicate multi-step signaling pathways leading to downstream effects. Under physiological conditions (left), NMDA receptor activation is regulated by magnesium (Mg^2+^) blockade, allowing controlled calcium (Ca^2+^) influx essential for synaptic plasticity and learning. In AD (right), chronic glutamate exposure and membrane depolarization lead to persistent Mg^2+^ displacement from the NMDA receptor channel. This results in receptor overactivation and uncontrolled Ca^2+^ influx. The sustained elevation of intracellular Ca^2+^ triggers downstream signaling cascades that activate necrotic and apoptotic pathways, culminating in permanent excitotoxic damage, synaptic loss, and neuronal death—key events in AD-associated neurodegeneration.

The role of zinc, discussed above, is particularly critical in regulating neuronal excitability and provides a direct link to the next key mechanism in AD: glutamate excitotoxicity. Over the past three decades, the excitatory neurotransmitter glutamate has been shown to cause several neurological diseases [98,99,100]. This pathological process, known as glutamate excitotoxicity (GNT), is a mechanism of cell death caused by excessive release of glutamate from neurons as well as glial cells. It was first described almost 50 years ago as a special type of region-specific lesion of nervous tissue, characterized by vacuolization of neurons in the hypothalamus of newborn mice [101]. GNT results from the binding of glutamate to its N-methyl-d-aspartic acid (NMDA) receptors [102], α-amino-3-hydroxy-5-methyl-4-isoxazolepropionic acid (AMPA) and kainate receptors, as well as metabotropic glutamate (mGlu) receptors [103]. Glutamate normally activates NMDA receptors, which regulate the entry of Na^+^ and Ca^2+^ into neurons. Under physiological conditions at rest, Mg^2+^ ions block the NMDA receptor channel, preventing excessive influx of these ions. In AD, due to a constant excess of glutamate, NMDA receptors are hyperactivated, Mg^2+^ loses its blocking effect and Ca^2+^ and Na^+^ enter the cell uncontrollably (Figure 7), which ultimately leads to an overload of the neuron with calcium, which triggers a chain of pathological reactions: oxidative stress and excitotoxicity [104,105,106]. Despite the proven role of excitotoxicity in neurodegeneration, its study is limited by two factors: (1) the insufficient effectiveness of existing drugs that affect neurotransmitter systems, and (2) the dominance of alternative hypotheses of pathogenesis. Moreover, excitotoxicity is a complex process closely associated with oxidative stress and impaired metal metabolism (especially zinc deficiency), which requires the development of combined therapeutic approaches [107]. The observed changes in the inhibitory neurotransmitter system, exemplified by γ-aminobutyric acid [108], and the ability of excitotoxicity to affect cognitive function earlier than Aβ and tau pathologies [106], suggest that excitotoxicity may have greater potential in the treatment of AD. Beyond intrinsic neuronal mechanisms, emerging research highlights the profound impact of systemic factors on AD pathogenesis, notably through the microbiota–gut–brain axis, which can directly influence central inflammatory and excitatory processes.

### 2.8. Microbiota–Gut–Brain Axis Hypothesis

**Figure 8:**
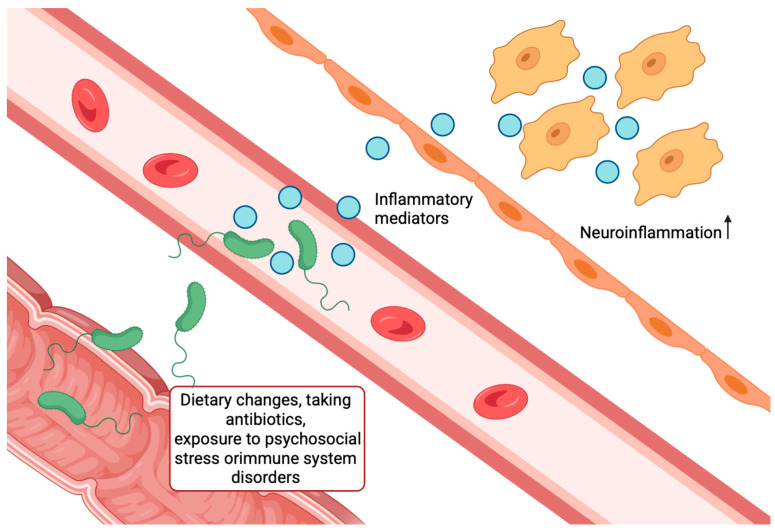
Impact of external factors on gut microbiota dysbiosis and neuroinflammation in AD. External triggers (diet, antibiotics, stress, immune disorders) disrupt gut microbiota balance, causing dysbiosis and loss of intestinal barrier integrity. This permits leakage of inflammatory mediators into circulation, promoting systemic inflammation and breaching the BBB. In the brain, these factors activate microglia and astrocytes, driving neuroinflammation that accelerates Aβ accumulation, tau pathology, and neuronal damage—creating a vicious cycle in AD pathogenesis.

The gut microbiota has been found to interact with the brain through the microbiota–gut–brain axis, regulating various physiological processes. In recent years, increasing attention has been paid to the influence of the gut microbiota on the development of the nervous system through this axis. It is believed that the intestinal microbiota regulates the development of the nervous system in three directions: immune, neural and endocrine-systemic, with intersections and interactions between them [109]. Changes in the host diet, antibiotic use, exposure to psychosocial stress, or immune system dysfunction can alter the relative proportions of bacterial species, leading to disruption of the composition and functionality of the microbiota in the form of dysbiosis (Figure 8) [110]. In turn, gut microbiota dysbiosis can lead to disruption of the intestinal barrier, resulting in a “leaky gut” [110] and facilitating pathogen entry into the portal and systemic circulation, leading to neuroinflammation in CNS disorders [111,112,113], such as depression or AD. In addition, the intestinal microbiota influences the development of the CNS through cell wall components and also regulates the production of cytokines through the systemic circulation [114]. The onset of systemic inflammation may result in inflammatory mediators crossing the BBB and affecting microglia, further exacerbating neuroinflammation [115]. This process is accompanied by a disruption of neurotransmission [116], which ultimately leads to degeneration and damage of neurons. Because gut microbiota can modulate neuroinflammation and neurodegenerative processes, its role in the development and progression of AD is poorly understood. The exact mechanisms by which the gut microbiome influences brain activity or its relationship to other pathophysiological features of AD remain unclear [117,118]. The neuroinflammatory and proteotoxic stress triggered by peripheral systems like the gut microbiota places a significant burden on the brain’s intrinsic quality control mechanisms, particularly the autophagic-lysosomal pathway, which is crucial for clearing dysfunctional proteins and organelles.

### 2.9. Abnormal Autophagy

**Figure 9:**
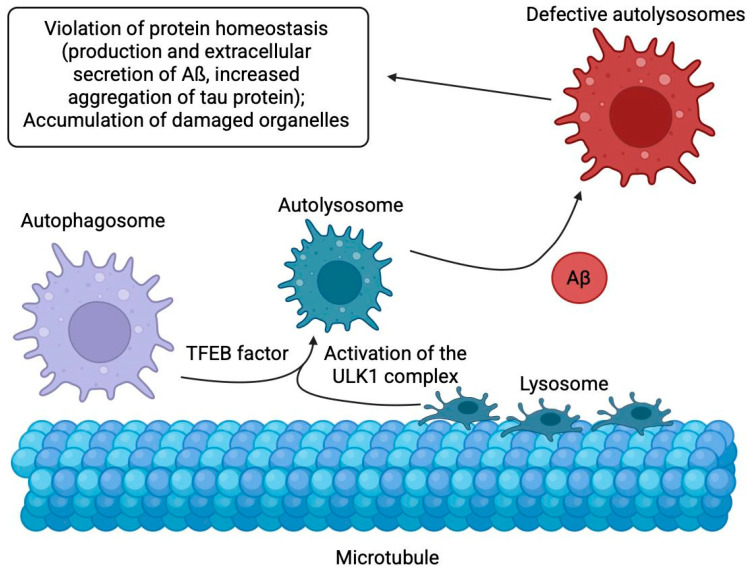
Dysregulation of the autophagic pathway in AD. The figure illustrates key impairments in this major cellular clearance mechanism. In AD, the initiation of autophagy (via ULK1 complex and TFEB activation) and the formation of autophagosomes are disrupted. This leads to the accumulation of defective autolysosomes, which fail to degrade their cargo. Consequently, there is a harmful buildup of Aβ peptides and hyperphosphorylated tau proteins, alongside damaged organelles. These accumulations contribute significantly to synaptic dysfunction and neuronal loss, hallmark features of AD pathogenesis.

Before Aβ accumulation in neurons, dysfunction of the endocytic pathway is observed, indicating its key role in pathogenesis [119], and this pathway integrates with the autophagic-lysosomal system to degrade and recycle proteins through a highly conserved cellular catabolic process called autophagy, where cytoplasmic material undergoes lysosomal degradation to eliminate long-lived proteins and organelles [120,121,122,123]. In mammals, autophagy occurs physiologically and enhances under cellular stress like protein accumulation, attempting to clear excess components through three main forms—macroautophagy (engulfing cargo via double-membrane autophagosomes for organelle and aggregate degradation), microautophagy (direct lysosomal engulfment via membrane invagination), and chaperone-mediated autophagy (selective translocation of motif-bearing proteins by chaperones like Hsc70)—with macroautophagy being most relevant in AD, triggered by ULK1 complex and TFEB activation [124]. These signals trigger the next stage—the formation of a double-membrane structure (phagophore), which “packages” the contents of the cell for processing [120]. In this case, the membrane source for phagophores can arise de novo from already existing intracellular structures such as the endoplasmic reticulum (ER), Golgi apparatus, mitochondria and plasma membranes [120,125]. Once closed, the fused structure, now called an autophagosome, delivers its contents to the lysosome via autophagosome-lysosome fusion, forming an autophagolysosome, where protein degradation occurs (Figure 9). While in healthy neurons autophagy completes the recycling of metabolites, in neurodegenerative diseases such as AD this process is disrupted. The accumulation of autophagic vacuoles in neurons associated with Aβ/APP-βCTF [126] indicates a clearance failure. This failure leads to disruption of protein homeostasis (production and extracellular secretion of Aβ, abnormal aggregation of tau protein) and accumulation of damaged organelles, such as dysfunctional mitochondria [127]. These disturbances not only confirm the key role of autophagy in the pathogenesis of AD, but also explain why its restoration is considered a promising therapeutic target. Autophagy-stimulating drugs, including small molecules and gene therapy, demonstrate neuroprotective effects in preclinical models of AD, making them promising therapeutic options. Moreover, studies in animals, cell models, and patients with late-onset SAD show that mitophagy defects exacerbate synaptic dysfunction and cognitive deficits, triggering a vicious cycle: Aβ and tau accumulation exacerbate oxidative stress and energy deficit, which in turn further suppress mitophagy.

Therefore, strategies aimed at improving mitochondrial function and stimulating mitophagy may slow down or prevent the neurodegenerative process in AD [127]. However, although interest in autophagic dysfunction in AD has certainly increased over the past few years, this area of research remains poorly understood. More research is needed to develop effective treatments for AD [128]. The progressive failure of autophagy to clear damaged cellular components not only contributes directly to proteotoxicity but also drives cells into a state of irreversible growth arrest known as cellular senescence, which has emerged as another fundamental mechanism in AD pathogenesis.

### 2.10. Cellular Senescence

**Figure 10:**
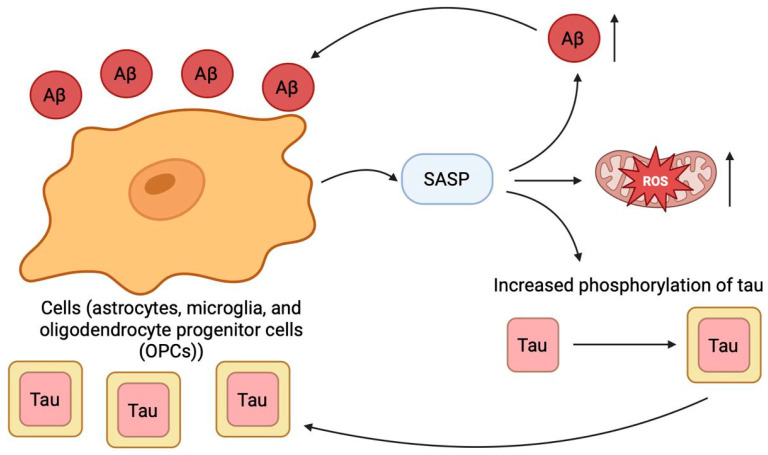
Pathological vicious cycle of cellular senescence in AD. In the AD brain, pathological factors such as Aβ accumulation and tauopathy drive astrocytes, microglia, and OPCs into a senescent state. These senescent cells secrete the SASP, which includes pro-inflammatory factors and ROS. The SASP exacerbates mitochondrial dysfunction, oxidative stress, and neuroinflammation, which in turn promotes further Aβ deposition and pathological hyperphosphorylation of tau. This creates a self-sustaining vicious cycle that amplifies both cellular senescence and AD pathogenesis.

Cellular senescence has emerged as a critically important process in the pathophysiology of AD, potentially acting as a bridge between aging, the primary risk factor for AD [129,130], and the core neuropathological hallmarks of the disease [131]. Growing evidence indicates that key glial cells, including astrocytes, microglia, and oligodendrocyte progenitor cells (OPCs), adopt a senescent phenotype in response to Aβ aggregates [132,133,134]. Beyond merely ceasing proliferation, these senescent cells actively secrete a plethora of pro-inflammatory and tissue-remodeling factors, known as the senescence-associated secretory phenotype (SASP) [131]. This SASP is not a bystander phenomenon; it is thought to create a toxic microenvironment that fuels neuroinflammation, disrupts extracellular matrix integrity [131], and propels the pathological progression of AD and other age-related neurodegenerative conditions [131,132,135]. A particularly detrimental aspect of this process is the establishment of a self-reinforcing pathological loop. Aβ and tau pathologies are potent drivers of cellular senescence [132,133,134]. In turn, the resulting SASP has been shown to exacerbate both Aβ deposition and tau hyperphosphorylation [131,132,135], thereby creating a vicious cycle that amplifies both neurodegeneration and further cellular senescence (Figure 10). This cycle is likely fueled by additional aging-related mechanisms, such as impaired proteostasis, accumulated DNA damage, and telomere dysfunction [136,137,138], which converge to induce both stress-induced and replicative senescence across diverse cell types in the AD brain, including not only glia but also neurons and vascular cells [139,140,141,142].

The functional consequences of this widespread senescence are severe. Affected cells lose their homeostatic functions, leading to a cascade of impairments: synaptic dysfunction, breakdown of the blood–brain barrier, failed remyelination, and a deficit in clearing toxic waste products [133,143,144,145]. In response, the field has developed therapeutic strategies termed “senotherapy,” which aims to either eliminate senescent cells (senolytics) or suppress the harmful SASP (senomorphics) [132,146,147,148,149]. Preclinical models offer promising evidence that senotherapy can mitigate AD-like pathology [132,150], positioning it as a novel and compelling therapeutic avenue [151,152]. However, the translation of these findings to the clinic faces significant challenges. The evidence for a direct causal role of senescence in human AD pathogenesis remains correlative, and the phenomenon appears highly heterogeneous across different brain cell types [139,140,141,142]. Therefore, future research must move beyond correlation to establish causality and unravel the distinct mechanisms by which senescent astrocytes, microglia, neurons, and endothelial cells each contribute to the disease process. This nuanced understanding is critical for developing targeted, effective, and safe senotherapeutic interventions for AD.

### 2.11. Endoplasmic Reticulum Stress

**Figure 11:**
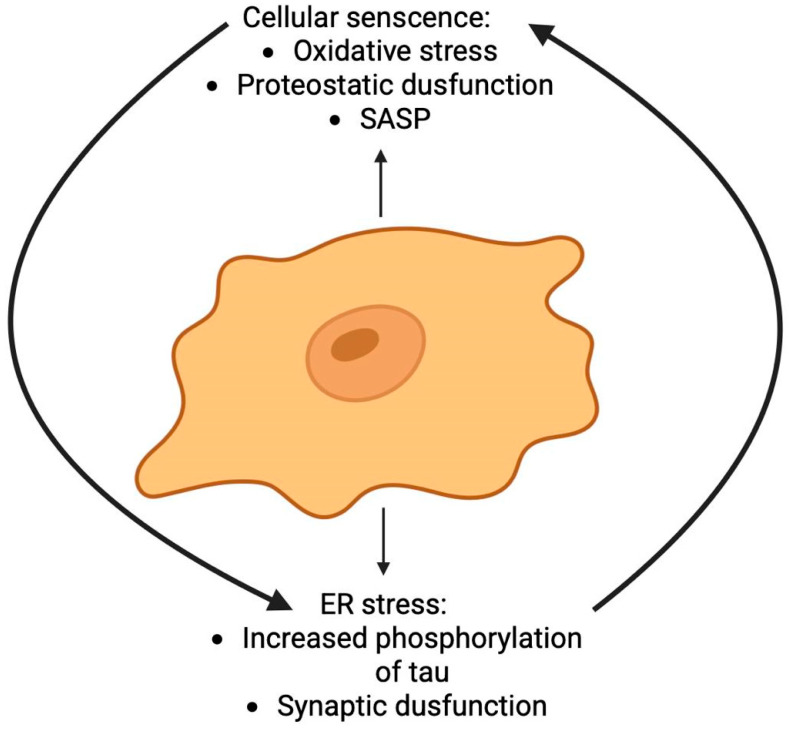
The synergistic interplay between cellular senescence and ER stress in AD pathogenesis. As illustrated, cellular senescence—driven by oxidative stress, proteostatic dysfunction, and SASP—creates a toxic milieu that induces ER stress. Critically, the SASP propagates proteotoxic stress and disrupts ER homeostasis in nearby cells, establishing a self-reinforcing feed-forward loop of dysfunction. This vicious cycle, depicted in the figure, culminates in tau hyperphosphorylation, synaptic failure, and neuronal death, ultimately accelerating disease progression.

The toxic microenvironment and proteostatic collapse driven by cellular senescence create ideal conditions for the activation of another key stress pathway in AD: ER stress (Figure 11). The pathological landscapes of cellular senescence and ER stress exhibit significant convergence in AD, suggesting a synergistic relationship that may profoundly accelerate disease progression. Common instigators, including oxidative stress and impaired proteostasis, serve as primary triggers for both processes. Critically, the SASP perpetuated by senescent cells (e.g., pro-inflammatory cytokines and ROS) can propagate proteotoxic stress and disrupt ER homeostasis in nearby cells, thereby establishing a feed-forward loop of dysfunction that links these pathways and amplifies neuronal damage [153]. Substantial evidence now positions ER dysfunction as a key mediator of neuro degeneration in AD a process likely worsened by the senescent milieu. Histopathological studies reveal a consistent co-occurrence of ER stress markers and pathological tau aggregates within vulnerable cortical regions of postmortem AD brains [154]. The proposed mechanistic cascade begins with the accumulation of misfolded proteins (notably Aβ and p-tau) and a concomitant loss of calcium homeostasis—conditions exacerbated by SASP factors. These disturbances foster an environment of heightened oxidative stress and metabolic dysfunction, which collectively converge to trigger severe ER stress. This, in turn, compromises synaptic integrity and promotes neuronal death. The involvement of specific stress kinases, such as PERK, eIF2α, and p38 MAPK, is strongly associated with the development of tau pathology, underscoring a molecular link between proteostatic collapse and neurodegeneration [153,154].

The core adaptive mechanism to this proteostatic failure is the unfolded protein response (UPR). An overabundance of misfolded proteins within the ER lumen prompts the activation of this sophisticated signaling network. This is initiated by the dissociation and subsequent activation of three key sensors—IRE1, PERK, and ATF6—from the chaperone BiP. While the immediate UPR aim is to restore equilibrium by halting global translation and upregulating protein-folding chaperones, its persistent activation under chronic stress conditions—such as that maintained by SASP-precipitates a decisive shift toward apoptotic signaling. The sustained upregulation of markers such as p-IRE1, p-PERK, ATF6α, and the pro-death transcription factor CHOP not only compromises neuronal survival but also exacerbates neuroinflammation, thereby intensifying the pathological milieu in AD [155].

The established role of ER stress in AD pathogenesis has galvanized the search for targeted therapeutics. Strategies aimed at modulating the UPR to alleviate ER stress and block its apoptotic signaling branch are under active investigation. A range of compounds, from repurposed pharmaceuticals to natural products like guanabenz, salubrinal, Tauroursodeoxycholic acid (TUDCA), and Chrysophanol have shown promise in preclinical models by effectively dampening the ER stress response, highlighting a viable pathway for therapeutic intervention [156].

In summary, the interplay between cellular senescence and ER stress represents a critical pathogenic nexus in AD (Figure 11). Their relationship is not merely concurrent but synergistic, creating a self-reinforcing cycle that drives proteostatic collapse, neuroinflammation, and ultimately, neuronal death. Targeting this axis, perhaps through combined senolytic and ER proteostasis-modifying strategies, may offer a novel therapeutic avenue to disrupt this vicious cycle and modify the course of AD.

### 2.12. Ubiquitin-Proteasome System

**Figure 12:**
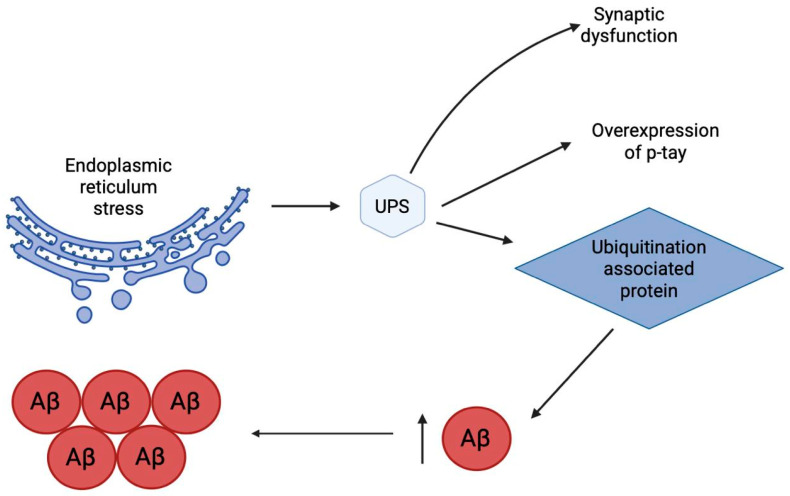
Dysfunction of the Ubiquitin-proteasome system (UPS) in AD. The diagram illustrates the cascade of pathological events: ER stress and proteostasis disruption lead to UPS impairment. This results in the accumulation of ubiquitinated proteins, impaired degradation of Aβ, and hyperphosphorylation of tau protein (p-tau). These processes synergistically exacerbate synaptic dysfunction and neurodegeneration, forming a vicious cycle in AD pathogenesis.

Disruption of proteostasis caused by endoplasmic reticulum stress naturally leads to dysfunction of the key system for degradation of misfolded proteins—the ubiquitin-proteasome system (UPS) (Figure 12). As the central effector of protein quality control, UPS impairment is a defining feature of AD pathology. Evidence positions the UPS not merely as a victim but as a crucial cytoprotective entity, whose progressive failure is a significant driver of AD progression [157]. Within the AD brain, this dysfunction manifests as a pronounced buildup of ubiquitin-conjugated proteins. This accumulation exacerbates the pathogenic cascade by interfering with the metabolism of Aβ, disrupting both its generation and clearance, and thereby promoting its aberrant aggregation. Furthermore, a robust mechanistic link connects UPS failure to the hyperphosphorylation and accumulation of tau protein [158]. These disruptions also contribute critically to the synaptic integrity loss and neuronal death characteristic of AD. The UPS executes its degradative function via a two-stage enzymatic process responsible for eliminating obsolete regulatory proteins and damaged soluble polypeptides [159]. Initial targeting occurs through ubiquitination, a covalent enzymatic reaction that tags a substrate protein with a chain of ubiquitin molecules. This tag then directs the marked protein to the 26S proteasome—a large multi-subunit complex—where it is unfolded, proteolyzed, and its constituent ubiquitin monomers are recycled. The ubiquitination cascade itself is a precise, three-tiered process involving a dedicated set of enzymes: ubiquitin-activating (E1), ubiquitin-conjugating (E2), and ubiquitin-ligase (E3) enzymes. The process culminates in an isopeptide bond formed between the C-terminal glycine of ubiquitin and a lysine ε-amino group on the target protein. E3 ligases are the pivotal determinants of substrate specificity within this system, governing the activation, function, and turnover of a vast array of proteins. The remarkable diversity of human E3s (approximately 800) enables exquisite control over cellular processes through their spatial, temporal, and substrate selectivity, as well as their ability to generate varied ubiquitin chain topologies (e.g., mono-, multi-, polyubiquitination). This system is dynamically regulated by deubiquitinating enzymes (DUBs), which catalyze the removal of ubiquitin, making the process reversible. The critical balance of ubiquitination and deubiquitination is essential for neuronal homeostasis [159] and its dysregulation is a recognized etiological factor in AD [160]. Given their central role, specific E3 ligases and DUBs are now understood to be key contributors to the pathological accumulation of both Aβ and tau (Figure 12). Consequently, pharmacological targeting of these enzymes represents a promising therapeutic avenue. Several E3-centric strategies have shown potential. For example, the m6A writer METTL3 has been found to facilitate the autophagic removal of phosphorylated tau by modulating the E3 ligase CHIP, resulting in improved AD phenotypes [161]. Similarly, sulforaphane-induced upregulation of CHIP has been proposed as a strategy to mitigate both Aβ and tau pathology [162]. Other approaches, like the natural compound geniposide, promote the degradation of the APP by enhancing the expression of the E3 ligase Hrd1 [163]. Notably, DUBs are increasingly viewed as superior pharmacological targets compared to E3 ligases. This is largely due to the structural challenges of targeting E3s, which often possess large, flat protein–protein interaction interfaces without deep catalytic pockets, making them difficult for small molecules to inhibit with high specificity and potency. In contrast, many DUBs have well-defined, druggable active sites with distinct catalytic residues, facilitating the rational design of selective inhibitors for personalized therapeutic interventions.

In conclusion, while E3 ligases and DUBs have emerged as fundamental regulators in AD, our understanding of their individual roles and therapeutic potential is still evolving. The future of UPS-targeted therapy lies in the precise identification of the specific E3s and DUBs that govern Aβ and tau proteostasis, followed by the development of highly specific activators or inhibitors. This refined approach holds immense promise for delivering effective, disease-modifying treatments for AD.

### 2.13. Comparative Analysis of Therapeutic Targets in AD

The diversity of pathogenic hypotheses for AD reflects the extreme complexity of the disease and raises the inevitable question of the comparative potential of different targets for therapy and diagnosis. Based on the presented review, a summary table (Table 1) has been compiled, ranking the key hypotheses based on their therapeutic promise, current level of clinical validation, and diagnostic utility. The analysis yields several key conclusions. First, targets with the highest level of clinical validation (amyloid and cholinergic hypotheses) demonstrate only moderate efficacy, highlighting the need to target earlier stages of the pathological cascade. Second, targets with high therapeutic promise (tau pathology, neuroinflammation, autophagy) are in early stages of development, and their successful application requires overcoming significant methodological challenges. Third, revolutionary advances in diagnostics (PET imaging, plasma p-tau, Aβ_42/40_ biomarkers) have been achieved thanks to fundamental discoveries in the amyloid and tau hypotheses.

Thus, it becomes clear that the future of AD treatment lies not in finding a single “correct” target, but in developing combined and personalized strategies. Such strategies should consider the disease stage, individual genetic profile, and pathobiological phenotype of the patient, simultaneously targeting several key links in the pathogenesis (e.g., amyloid removal + suppression of tau pathology + modulation of neuroinflammation + support of proteostasis).

## 3. Targeted Therapeutic Agents for AD

Given the complexity and multifactorial nature of AD pathogenesis, the development of effective therapeutic agents requires precise targeting of the key molecular targets discussed earlier. Currently, organic ligands capable of selectively interacting with such targets, modulating their activity or blocking pathological processes, are being actively studied. Depending on their structure and mechanism of action, these compounds can be divided into several classes: small molecules, peptides, antibodies (and their fragments), natural ligands, and hybrid multifunctional molecules. Each of these classes has unique physicochemical and pharmacological properties, which determines their advantages and limitations in the development of radiopharmaceuticals and other innovative approaches to AD therapy. The key characteristics of these therapeutic agents are summarized in (Table 2). In the following subsections, we will take a closer look at the characteristics of each type of compound and their potential in treating this disease.

### 3.1. Small Molecules

Small molecules represent a promising tool for targeted therapy of AD due to their versatility and potential for chemical modification. A striking example is methylene blue (MB), which not only penetrates the BBB but also reduces tau protein aggregation. Bis(hydromethanesulfonate) leuco-methylthionine ion (LMTM), a modified MB, has been shown to be effective in preclinical studies. Initial clinical studies demonstrated safety and good pharmacokinetics but did not result in significant improvement in disease [164]. However, long-term monotherapy with LMTM in a large clinical trial showed improvement in brain atrophy and cognitive function. Nitrocatechols were tested for their ability to modulate tau protein aggregation and 5-nitro-α-cyanocarbonamide derivatives of caffeic acid and phenylethyl ester of caffeic acid were found to have good antiaggregatory activity [165]. A hybrid molecule of naphthoquinone and dopamine, NQ-DA, was developed to combat tau protein aggregation [166]. The molecule targets PHF(VQIVYK) and PHF*(VQIINK) motifs and effectively inhibits tau protein aggregation. Molecules targeting multiple kinases are predicted to be a rational strategy to prevent hyperphosphorylation. A series of diaminothiazole screens identified LDN193594, which inhibits CDK5 and GSK3β activity with an IC50 of 1 nM [167]. In vivo evaluation in transgenic (Tg) mice showed that LDN193594 is nontoxic, reduces hyperphosphorylated tau tangles, and improves cognitive function compared to controls. A new molecule, SCR1693, based on tacrine, was also developed that promotes tau protein dephosphorylation and reduces Aβ production [168]. As discussed previously, neuroinflammation, in particular microglial activation, plays a key role in disease progression. To quantify this process in vivo, the ligand (R)-[^11^C]PK11195(1-(2-chlorophenyl)-N-methyl-N-1(1-methylpropyl)-3-isoquino linecarbonamide) is used, which selectively binds to the TSPO protein on the surface of activated immune cells. The small molecule D-APV (D-AP5), a selective NMDA receptor blocker, was also shown to completely prevent the uptake of toxic Aβ_1–42_ (at a concentration of 15–30 μM) by brain cells. As a result, there was no increase in the level of the destructive enzyme cathepsin D and no activation of microglia. These effects have been demonstrated in experiments on live hippocampal slices [169]. Caffeic acid phenylethyl ester (CAPE) is a potent antioxidant and anti-inflammatory agent that has been shown to be effective in combating Aβ oligomers and their associated toxicity in a Tg mouse model [170]. Treatment induces Nrf2 expression and restores memory and cognitive function. Gut dysbiosis has pathological consequences for the gut–brain axis and is considered a potential therapeutic target [171]. Multifunctional molecules were developed by conjugating clioquinol (Clq) moiety for metal chelation and polyphenolic moiety EGCG as antioxidant module to create Aβ aggregation modulator TGC86, which effectively suppresses amyloid aggregation and mitochondrial damage [172]. Emerging targeted strategies also focus on modulating neural signaling and brain metabolism. Phosphodiesterase (PDE) inhibitors have garnered significant attention for their potential in AD therapy. A prominent example is PF-04447943, a potent and selective phosphodiesterase 9A (PDE9A) inhibitor. Its mechanism of action involves elevating guanosine 3′,5′-cyclic monophosphate (cGMP) levels in the brain and cerebrospinal fluid. Preclinical evidence demonstrates that PDE9A inhibition enhances synaptic plasticity, improves memory in cognitive models, and prevents the reduction in dendritic spine density in transgenic mice overexpressing APP, such as the Tg2576 model, which results in high levels of Aβ production [173,174]. In parallel, the field is increasingly exploring the strategy of drug repurposing, seeking multifunctional agents that can simultaneously address several pathological hallmarks. Another novel approach involves the repurposing of Sodium-Glucose Cotransporter-2 (SGLT2) inhibitors, commonly used for type 2 diabetes [175]. Beyond their well-established glycemic benefits, these agents exhibit direct neuroprotective effects, including reduced neuroinflammation and improved cerebral metabolism, which may underpin the potential of gliflozins to improve cognitive function in patients with both diabetes and AD [176]. Emerging clinical evidence suggests that SGLT2 inhibitors may be associated with a lower risk of dementia and slower cognitive decline, positioning them as promising multifunctional agents for AD therapy [177]. In addition to multi-target approaches aimed at the main pathological features of AD, modern therapy increasingly turns to targeting fundamental mechanisms of aging, one of which is cellular senescence. For example, Bcl-2 family inhibitors, BH3 mimetics, Hsp90 inhibitors, p53 binding inhibitors, HDAC inhibitors, p38MAPK inhibitors, and JAK/STAT inhibitors are widely used senolytics (Table 2) [148,150,178,179,180,181,182,183]. Another promising approach involves CSF1R inhibitors such as GW2580, which specifically target microglial function. Preclinical studies demonstrate that GW2580 alleviates Aβ accumulation as well as neuritic and synaptic damage by modulating microglia [184]. Furthermore, genetic approaches to senescent cell clearance have shown significant promise. The inducible elimination of p16+ senescent cells using the p16-3MR model and treatment with AP20187 improved cognitive function in preclinical models, demonstrating proof-of-concept for whole-body senescent cell clearance as a therapeutic strategy for AD [185]. Senolytics significantly alleviate AD pathology [132,150]. For instance, dasatinib (a protein tyrosine kinase inhibitor) and quercetin (a natural compound that serves as a senomorphic) co-administration effectively removes senescent OPCs, alleviates neuroinflammation, attenuates Aβ accumulation, and improves cognitive function, suggesting the great potential of senescent cell clearance in AD therapy [132]. In addition to senolytics, which physically remove senescent cells, there are senomorphics, molecules that inhibit or block some characteristics of SASP without killing the senescent cells themselves [150]. Senomorphic therapy involves two main strategies: targeting SASP-associated pathways (such as PI3k/Akt, JAK/STAT, and mTOR) and transcription factors (NF-κB, C/EBPβ, STAT3), and neutralizing SASP factors, including inflammatory cytokines [186]. Among the most studied senomorphics is rapamycin, which reduces cellular senescence primarily by inhibiting the mTOR signaling pathway [187,188]. Although many studies have demonstrated beneficial effects of rapamycin on AD pathology, including reduction in Aβ accumulation [189], tau hyperphosphorylation [190], and neuroinflammation [191], as well as improvement of cognitive function [192], its effects remain controversial [193]. Another widely studied senomorphic is metformin, which was originally developed for the treatment of type 2 diabetes [188]. Metformin attenuates cellular senescence and SASP by inhibiting NF-κB nuclear translocation and shows potential in the treatment of AD [194,195]. Clinical data suggest that metformin may reduce the risk of AD in elderly patients with diabetes and have a beneficial effect on patients with cognitive impairment [167,196,197,198]. In addition to targeting pathological proteins and cellular senescence, targeting the ubiquitin system is a promising direction. DUBs, such as USP25, play an important role in regulating the stability of key proteins involved in the pathogenesis of AD. Moreover, the catalytic groups of certain DUBs are significantly different, which makes it possible to develop specific DUBs inhibitors for personalized treatment of AD. miRNA-455–3, which owns a strong capacity of binding to USP25, has been identified as a possible AD peripheral biomarker [199], and its high expression has been validated in fibroblasts and B lymphocytes from AD patients. Up to date, several DUB inhibitors have been developed, including LDN-57444, RP-619, HBX41108, and FT3967385, which have shown efficacy in regulating various cellular processes relevant to neuronal health and survival. Nevertheless, further investigation is necessary to completely grasp the effectiveness of these inhibitors in AD. Other promising senomorphics include: NF-κB inhibitors [186,194], ATM inhibitors (e.g., KU-55933 and KU-60019), which reduce the expression of senescence markers and SASP levels [149,200] and p38MAPK inhibitors (such as SB203580 and BIRB796), which attenuate the signs of cellular senescence and reduce the content of SASP components, mainly by enhancing the transcriptional activity of NF-κB [148].

There are many more small molecules capable of selectively interacting with key targets that could be used as diagnostic agents for the treatment of AD [201,202], but their effectiveness is limited by delivery issues.

### 3.2. Peptides

Peptides and oligonucleotides are superior to small molecules as delivery systems due to their biodegradability, biocompatibility, low toxicity, ease of synthesis, and ability to control composition [203,204,205]. A prime example is RVG29, a 29-amino acid peptide derived from the rabies virus glycoprotein (RVG), which demonstrates these benefits in practice. It specifically binds to nicotinic acetylcholine receptors (nAchR) in neurons [203]. Importantly, even unmodified RVG29 on the surface of exosomes delivered siRNA against BACE1 into mouse neurons and reduced BACE1 expression by 60% and Aβ levels by 55% in AD [206]. Another example is GHK (glycyl-l-histidyl-l-lysine), a peptide released from secreted protein rich in cysteine (SPARC), which, through proteolytic degradation, has shown that GHK can improve TGFβ1 signaling [207], a pathway associated with Aβ deposition and NFT formation [208]. In addition, GHK has been shown to be an endogenous antioxidant, reducing hydroxyl and peroxyl radical levels [209], thereby improving cognitive performance in aging mice [210,211]. The natural tripeptide glutathione (GSH, γ-l-glutamyl-l-cysteinyl-glycine), which plays a key role in antioxidant protection, has a similar mechanism of action. In AD, brain GSH levels are reduced, leading to significant oxidative stress, and therefore increasing GSH levels is a rational approach to treating AD. N-acetyl-l-cysteine (NAC) treatment is known to increase GSH levels and have an antioxidant effect. NAC treatment in aging rats increases antioxidant enzyme levels [212]. In the work of Eisenberg and co-authors, the tau protein aggregation inhibitor D-TLKIVW was developed, which is a peptide consisting entirely of D-amino acids [213]. The designed peptidomimetics consisting of a pentapeptide with two delta-linkages of ornithine and a lower chain consisting of 2 residues and a β-helical peptidomimetic of the “Hao” structure adopt a β-helical conformation and effectively suppress the aggregation of peptides with a PHF motif [167]. Aβ motif-derived fusion peptides KLVVF, P4 and P5, retained tau in a random coil state as detected by CD spectroscopy, inhibiting tau aggregation and rescuing Neruro2a cells from tau toxicity [167]. The E3 ubiquitin ligase-specific peptide ALAPYIP was developed based on the von Hippel-Lindau (VHL) tumor suppressor protein, a natural E3 ligase substrate. This peptide mediates ubiquitin-dependent degradation of tau, reducing its levels in both primary neurons and a transgenic mouse model of AD [214]. Peptide-based molecules have anti-aggregation properties and suppress fibrillogenesis. Soto et al. developed and tested the pentapeptide iAβ5, which demonstrated the ability to inhibit Aβ aggregation and dissolve formed fibrils both in vitro and in vivo [215]. Ongeri and colleagues reported hairpin peptide mimetics containing piperidine-pyrrolidine moieties to inhibit Aβ aggregation [216].

### 3.3. Antibodies and Their Fragments

Bapineuzumab was the first monoclonal antibody developed against the N-terminal region of Aβ_42_, which selectively binds to oligomers and fibrils. Phase III clinical trials showed a weak therapeutic effect, indicating the limited effectiveness of this antibody and the need to find more effective immunotherapeutic agents [217]. Solanezumab was designed to target the Aβ_13–28_ segment, which has been shown in preclinical studies to be safe and effective in clearing Aβ from the brain. Clinical studies have shown that antibody treatment is safe and results in a dose-dependent reduction in Aβ levels, but does not improve memory impairment [218]. Gantenerumab is another monoclonal antibody designed to target the N-terminal and central regions of Aβ. Clinical trials have yielded mixed results in terms of therapeutic benefit and safety [219]. Crenezumab is an immunoglobulin G (IgG) monoclonal antibody that binds to oligomers, fibrils, and plaques, inhibiting Aβ aggregation and disrupting fibrils [220]. Aducanumab, an antibody targeting a conformational epitope of Aβ that binds to fibril aggregates, has received conditional approval from the FDA for the treatment of AD. Clinical trials have demonstrated beneficial effects in terms of improved cognitive performance [221]. The monoclonal antibody DC8E8 identified an epitope spanning amino acid residues 294–305 that is essential for pathogenic interactions between tau and itself. The peptide vaccine AADvac1 was derived from this region (KDNIKHVPGGGS) and tested in a transgenic rat model [222]. In phase II clinical trials, the vaccine was found to be safe [223], but did not improve cognitive function. An antibody to the tau protein, C2N-8E12 (ABBV-8E12), was also developed that reduces tau protein levels and improves cognitive impairment [224]. Phase I clinical trials have shown safety, good pharmacokinetics and brain penetration with low immunogenicity. BIIB092 (gosuranemab, BMS-986168) is a monoclonal antibody that selectively targets the N-terminal fragment of tau protein [225]. Preclinical studies are promising, and clinical safety studies have shown that the drug is well tolerated at doses up to 2100 mg. Treating mice with the Tg mutation with the monoclonal antibody Ta1505, developed against pSer413, reduced tau protein levels and improved synaptic density and cognitive function [226]. Preclinical studies of two tau-specific monoclonal antibodies—43D (epitope 6–18) and 77 × 10^9^ (epitope 184–195)—demonstrated a significant reduction in tau protein levels and complete restoration of cognitive functions [227]. The use of monoclonal antibodies is showing promising results in early stages of clinical trials. The success of therapy in the treatment of AD stages III and IV remains to be seen. It is important to note that the therapeutic application of monoclonal antibodies extends beyond targeting protein aggregates to also modulate the inflammatory microenvironment of the aging brain. For example, monoclonal antibodies against IL-6, NF-κB, and IL-8 effectively alleviate chronic inflammation induced by SASP [186,228]. However, this senomorphic approach has drawbacks, such as the need for chronic administration to achieve an effect and the potential inhibition of pathways important for maintaining tissue homeostasis [150].

In conclusion, the experience with monoclonal antibodies against Aβ and tau protein has shown that targeting only one pathological feature of AD brings limited benefit. This confirms that AD is a multifactorial disease requiring a combined approach. A promising direction is the combination of senotlytics agents (removing Aβ and tau protein) with senomorphic ones (suppressing neuroinflammation), which will allow simultaneous action on several key links in pathogenesis to achieve a significant therapeutic effect.

### 3.4. Natural Ligands

Natural ligands in AD therapy are used as independent therapeutic agents. However, it is important to emphasize that while many natural compounds show compelling promise in preclinical studies, their clinical efficacy remains largely unproven, with most demonstrating weak or mixed results in human trials. A striking example of natural molecules are vitamin E and selenium—these are natural bioavailable antioxidants that demonstrate high bioavailability and a pronounced neuroprotective effect in experimental models in vitro and in vivo. However, in studies with AD patients, individual nutritional supplements and combinations of supplements show only minimal improvement with mixed overall results [229], illustrating the translational challenge common to many natural compounds. A recent study found that patients whose diet included flavonols had a lower risk of developing AD [230]. These results indicate the possible therapeutic potential of flavonols and the need for further study of the effects of dietary flavonols in the treatment of AD. This group of flavonols also includes Luteolin, which has been shown to inhibit Aβ and tau protein aggregation and has potent anti-inflammatory and antioxidant properties, making it a promising candidate for AD therapy [156]. Resveratrol, a natural antioxidant, has demonstrated beneficial effects in many in vitro and preclinical studies, though clinical trial results have been inconsistent, underscoring the translational challenges in this field [231]. Fisetin was evaluated for its antioxidant and anti-inflammatory effects in an aging rat model [232]. The reduction in oxidative stress and neuroprotective effects of this compound make fisetin a potential candidate for an anti-AD drug. Like most natural compounds discussed here, fisetin requires rigorous clinical validation to establish its therapeutic utility in AD. While fisetin exerts its anti-AD efficacy through oxidative stress reduction and neuroprotective effects, acitretin, a synthetic vitamin A derivative, acts differently by activating α-secretase and enhancing non-amyloid processing of APP. Initial clinical study showed safety and an activating effect as evidenced by increased cerebrospinal fluid sAPP levels and decreased Aβ levels [233]. Seaweed-derived sodium oligomannate has been shown to inhibit Aβ aggregation and restore healthy gut microbiota [234]. Coconut oil is a source of ketone bodies, which can directly provide energy to cells. A randomized controlled trial found that a Mediterranean diet enriched with coconut oil improved cognitive function in patients with AD [235]. The bile acid TUDCA exhibits neuroprotective effects by inhibiting apoptosis, reducing oxidative stress and mitochondrial dysfunction, and promoting Aβ clearance [130]. It is currently in clinical trials for the treatment of AD. Curcumin derivative PE859, designed to target both Aβ and tau aggregation, effectively reduced Aβ and tau levels and improved cognitive function in a mouse model [236]. Notably, clinical evidence for curcumin itself has been largely disappointing despite strong preclinical data, illustrating the recurrent theme of limited translational success with natural compounds. Ginkgo biloba extract EGb 761 is widely used to treat neurological disorders, including AD. Studies have shown that EGb 761 can significantly improve cognitive function, neuropsychiatric symptoms and daily activities in patients with mild to moderate dementia, and alleviate symptoms in patients with mild cognitive impairment [237]. Ginkgolide A, another compound derived from Ginkgo biloba, attenuates Aβ-induced abnormal depolarization and inhibits NMDA receptors [238]. Several natural ligands and plant extracts are acetylcholinesterase inhibitors that may be used to treat AD. Two benzophenanthridine alkaloids from Zanthoxylum rigidum root extract, namely nitidine and avicin, showed dual inhibition of acetylcholinesterase and butyrylcholinesterase, and demonstrated moderate antiaggregatory activity against Aβ_42_ and inhibition of monoamine oxidase A [239]. Chrysophanol, a natural anthraquinone, also exhibits AChE and BACE1 inhibitory activity and reduces neuroinflammation, indicating its multifaceted therapeutic potential [130]. Helminthosporin, an anthraquinone isolated from *Rumex abyssinicus* Jacq., showed dual inhibitory effects on acetylcholinesterase and butyrylcholinesterase, as well as high BBB permeability [240]. These properties indeed confirm that natural ligands represent a promising platform and open new possibilities for targeted therapy of AD, combining targeted delivery with multifunctional therapeutic action. While these properties suggest natural ligands represent a promising platform for AD therapy, the field faces substantial challenges in translating multifaceted mechanistic actions into clinically effective treatments. The gap between promising preclinical results and demonstrated clinical efficacy remains a critical hurdle that requires more rigorous clinical investigation and better understanding of bioavailability, optimal dosing, and standardization of natural products.

### 3.5. Hybrid Multifunctional Molecules

Analysis of the results of structural and functional analysis of vector molecules formed the basis for the development of highly effective compounds through their hybridization, functionalization and conjugation into a single molecular structure. A powerful nanoparticle α-secretase activator, APH-1105, developed on this principle has been developed for intranasal use in AD and is undergoing clinical trials. Safety and efficacy assessed in phase 2 clinical trials will help determine the potential of the candidate drug in the near future (NCT03806478) [241]. Lee et al. developed a novel small molecule compound ML obtained by integrating structural components for controlling Aβ aggregation, metal chelation, ROS regulation, and antioxidant activity [242]. ML reduces Aβ and Aβ-metal toxicity, decreases ROS levels, and has BBB permeability. Another small molecule, DMPD, was created by modifying the redox properties to redirect Aβ to a non-toxic aggregation pathway via covalent adduct formation [243]. Detailed biochemical, biophysical and molecular dynamics studies have shown that adduct formation between the ligand and the peptide occurs through intramolecular cross-linking dependent on primary amines. DMPD treatment in the 5xFAD Tg mouse model significantly reduced Aβ levels and reversed memory impairment. DMPD treatment in the 5xFAD Tg mouse model significantly reduced Aβ levels and reversed memory impairment [243]. Ber-D with polyphenolic groups has better chelating and antioxidant properties and reduces toxicity by reducing the interaction of Aβ aggregates with the mitochondrial membrane [244]. Ber-D exhibits antioxidant properties in vitro by reducing ROS and oxidative stress, preventing DNC and protein damage, and protecting PC12 neuronal cells from Aβ-induced toxicity and apoptosis. A highly potent theranostic agent was created by combining a distyrylbenzene moiety with metal-chelating triazamacrocycles and antioxidant vanillyl groups that reduces Aβ aggregates, tau protein and activates microglia [245]. Such rationally designed, highly potent molecules have great potential as future treatments for AD.

## 4. Radiopharmaceuticals for the Treatment and Diagnosis of AD

Despite the active search for therapeutic agents that target multiple AD targets (reviewed in the previous sections), early and accurate diagnosis remains a critical challenge and effective therapy remains an unmet need. Among all targets, Aβ and tau are the most specific histopathological markers of AD and key drivers of neurodegeneration, making them prime targets for radiopharmaceuticals.

In this section, we focus on the current development and application of Aβ- and tau-specific radiopharmaceuticals, discussing their design, binding mechanisms, advances in clinical imaging, and prospects for targeted radionuclide therapy of AD.

### 4.1. Radiopharmaceuticals for Aβ Imaging

**Figure 13:**
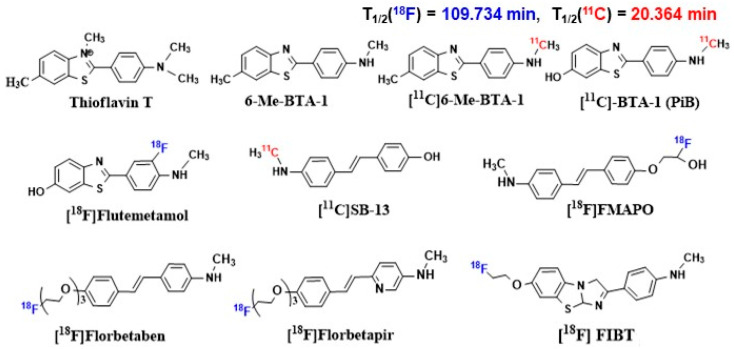
Chemical structures of historical and current Aβ-specific PET ligands for imaging, presented in order of their emergence and clinical adoption. The radionuclides shown are fluorine-18 (T_1_/_2_ = 109.734 min) and carbon-11 (T_1_/_2_ = 20.364 min).

Early and accurate detection of Aβ in vivo has been made possible by the development of specific PET ligands. Their story began with thioflavin T (ThT), a dye that can bind to amyloid fibrils in vitro [246]. Although ThT is not used therapeutically due to its inability to cross the BBB, it laid the foundation for the development of modern ligands that combine high affinity for Aβ or tau protein with optimized pharmacokinetic properties. The evolution of these compounds reflects a series of strategic trade-offs between affinity, pharmacokinetics, and practical applicability, rather than a straightforward linear progression. Because of this major drawback, scientists began developing new, more lipophilic derivatives of the dye, such as 6-Me-BTA-0, 6-Me-BTA-1, and 6-Me-BTA-2. These new compounds effectively stained plaques in tissue samples and bound to them with high affinity. However, one of them (6-Me-BTA-1) had its own problem: it did not exhibit the necessary fluorescent changes (spectral shifts) upon binding to the plaques, which is absolutely crucial for an effective fluorescent probe [247]. However, this issue became irrelevant when scientists decided to use the same molecule for a different type of diagnostics—PET imaging. When they developed a radioactive tracer based on its structure, it led to a major breakthrough. Further modification of the structure, namely the introduction of a radioactive label [^11^C] at position 6 of the benzothiazole ring of BTA-1. [^11^C]6-Me-BTA-1 turned out to be the best in this series. It was 6-fold more lipophilic, readily penetrated the BBB in rodent brain, and bound 44-fold more strongly to synthetic Aβ fibrils than Th-T [247,248]. These properties were confirmed by multiphoton microscopy on its unlabeled analogue BTA-1 [249]. The creation of [^11^C]-BTA-1 and its derivative, the Pittsburgh compound (PiB), synthesized by a group of scientists from Pittsburgh, was a breakthrough that allowed for the first non-invasive detection of Aβ plaques during a patient’s lifetime. Unlike Congo red, PiB combines high affinity for Aβ with the ability to penetrate the BBB and be rapidly cleared metabolically, making it the “gold standard” in PET diagnostics of AD [250]. The success of PiB has stimulated the development of a new generation of small molecules. However, a critical limitation of PiB is the short 20 min half-life of Carbon-11, which restricts its use to major research centers with an on-site cyclotron and limits imaging protocols The need for wider clinical utility spurred the creation of Fluorine-18-based ligands (t½ = 110 min) a broader class of compounds whose key characteristics are compared in (Figure 13). While solving the half-life issue, this adaptation introduced a new trade-off. [^18^F]-radioligands have been developed and evaluated both in vitro [251] and preclinically, of which [^18^F]Flutemetamol, also known as [^18^F]GE067 ([^18^F]3′F-PiB), was selected [252]. In vivo studies in rats and mice have shown that it has similar pharmacokinetics to PiB. Both drugs penetrate the brain readily, but [^18^F]Flutemetamol, a PiB analog, which is more lipophilic, was cleared from the brain approximately 1.4 times more slowly, particularly from white matter [253]. These compounds retained high affinity for Aβ, but due to the longer half-life of [^18^F] (110 min vs. 20 min for [^11^C] in PiB) they became more practical for clinical use [254]. To reduce the lipophilicity of the ligands, the stilbene derivative [^11^C]SB-13 (4-methylamino-4′-hydroxystilbene) was discovered. Early attempts to develop [^18^F]-labeled SB-13 were unsuccessful due to the high lipophilicity and non-specific binding to the brain exhibited by [^18^F]SB-13 derivatives with a fluoroalkyl group at both ends of the structure. Therefore, the stilbene framework was further modified by introducing various functional groups. Based on in vitro and in vivo biological studies, a NH-CH_3_ [^18^F]FMAPO derivative with a 2-fluoromethyl-1,3-proplenodiol group attached to the phenolic end of the molecule was selected. This compound demonstrated not only selective and specific binding to Aβ plaques in AD, but also a significantly higher rate of brain penetration—achieving almost three times the levels of [^18^F]Flutemetamol in half the time (2 min vs. 5 min) [251,255]. To circumvent the in vivo metabolic complications that may arise due to the presence of a chiral center in the fluorine-containing side chain, another series of stilbene derivatives were synthesized with polyethyleneglycol units of different lengths (*n* = 2–12) attached to the 4′-hydroxyl group, with ^18^F attached to the end of the polyethyleneglycol side chain. This also allowed for the maintenance of low molecular weight, adjustment of lipophilicity, and facilitation of ^18^F-labeling via nucleophilic substitution. Structure-activity relationship studies showed that high binding affinity was maintained at *n* < 8. In vivo biodistribution studies have shown a marked decrease in brain penetration at *n* > 5 [256,257,258], possibly due to an increase in molecular weight and total surface area. Of the four ligands that performed well in in vitro and in vivo studies, [^18^F]Florbetaben, also known as AV-1 or BAY94-9172, was selected with *n* = 3. Although [^18^F]Florbetaben did not have the highest affinity for Aβ compared to its structural analogs or the highest washout rate from healthy mouse brain [257], it nevertheless demonstrated selectivity for Aβ and negligible binding to NFTs, Pick bodies, Lewy bodies, and glial cells [255]. Preclinical studies in various animal species have shown that [^18^F]Florbetaben does not cause significant side effects at doses corresponding to 100 times the expected clinical dose [259]. The development of [^18^F]Florbetapir also known as [^18^F]AV45 [259] demonstrated a 2-fold higher binding affinity for Aβ in AD compared to [^18^F]Florbetaben but is characterized by slower accumulation kinetics and brain half-life [257,260]. This underscores that the “best” ligand is not defined by a single parameter but by its suitability for a specific clinical or research purpose. In scientific studies, the best imidazobenzothiazole derivative [^18^F]FIBT was described by Yousefi et al. and was named the first high-contrast Aβ imaging agent along with [^18^F]Florbetaben. It also demonstrated excellent pharmacokinetics, selectivity and high affinity for Aβ fibrils in vitro and in vivo, comparable to the gold standard PiB [261,262]. However, its complex synthesis and the current lack of extensive clinical data represent significant barriers to widespread adoption, a common challenge for promising pre-clinical agents. Despite these hurdles, the evolution in the development of Aβ-PET ligands, as visualized in (Figure 13) and (Table 3), has been substantial. High affinity for Aβ (as in [^18^F]FIBT) is often compensated by complex synthesis, while the practicality of [^18^F] ligands (due to their long half-life) may be accompanied by slower kinetics and non-specific binding. This progress has profound clinical implications: it not only enables non-invasive diagnosis but also allows for the precise monitoring of disease progression and the assessment of therapeutic efficacy in anti-amyloid trials. It is likely that new-generation Aβ-PET imaging will improve the signal-to-noise ratio, making it more suitable for quantifying disease progression and therapeutic monitoring. Although the radiopharmaceuticals described above have demonstrated reliability in detecting AD pathology, the tau protein is becoming an increasingly important target over time.

### 4.2. Radiopharmaceuticals for Tau Imaging

**Figure 14:**
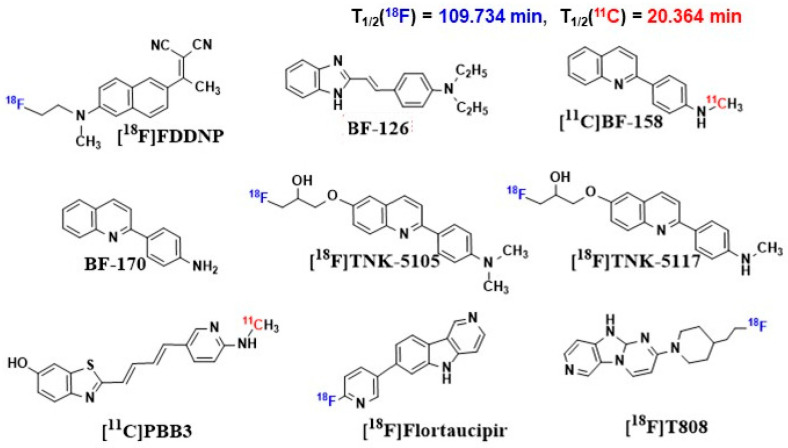
Chemical structures of historical and current tau-specific PET ligands for imaging, presented in order of their emergence and clinical adoption. The radionuclides shown are fluorine-18 (T_1_/_2_ = 109.734 min) and carbon-11 (T_1_/_2_ = 20.364 min).

The evolution of tau-PET ligands is characterized by incremental advances, with each novel compound overcoming specific prior limitations yet often exhibiting new drawbacks. ^18^F-FDDNP was the first PET ligand to enable visualization of tau pathology in vivo. However, subsequent studies have found its cross-reactivity with Aβ plaques due to the structural similarity of the β-sheet conformations in NFTs and amyloid deposits [263]. Benzimidazole and quinoline derivatives BF-126, BF-158/[^11^C]–BF-158, and BF-170 became the first selective radioligands for tau-PET [264]. They have good pharmacokinetics but insufficient specificity. Their optimization led to the creation of [^18^F]-THK5105 and [^18^F]-THK5117, which, despite improved binding to PHF-tau, continue to interact with myelin, raising questions about the validity of signals in areas like the temporal stem [265,266,267]. [^11^C]-PBB3 exhibits selective binding to pathological tau aggregates in AD and other tauopathies, with a 40-fold affinity advantage over Aβ [268], marking a breakthrough in selectivity; however, its major limitations—a short half-life and metabolism leading to brain-penetrating metabolites—pose significant challenges for accurate quantification and widespread clinical use [269]. Perhaps the most telling example of this trade-off is the development of Flortaucipir ([^18^F]-T807, also known as [^18^F]-AV1451) and [^18^F]-T808 which demonstrated high affinity and selectivity for tau protein over Aβ, along with favorable pharmacokinetic properties such as good brain uptake and rapid clearance [270]. However, [^18^F]-T808 had a significant drawback—in vivo defluorination, leading to the accumulation of radioactive fluorine ([^18^F]) in the bones of the skull [271]. Flortaucipir, in turn, not only showed a distribution consistent with pathological tau protein deposition in AD, but also became the first tau PET ligand approved by the FDA (2020) for the diagnosis of this disease. A critical analysis of current tau-PET ligands (Table 4) reveals a clear pattern: each new drug addresses certain problems of its predecessor, but often introduces new drawbacks. This creates an imperative for continued development and rigorous validation of imaging methods in clinical trials. The structural formulas of the described tau-PET ligands are presented in (Figure 14).

As detailed in (Table 4), a clear pattern emerges: each ligand carries a unique set of limitations that must be carefully considered. These limitations have a twofold implication. First, they drive the continuous imperative to develop more specific and reliable diagnostic agents. Second, and more critically for therapeutic development, they necessitate those clinical trials using these biomarkers rigorously validate their imaging protocols to account for these shortcomings. Consequently, the field is stimulated to actively search for novel agents with an improved profile.

Consequently, the field is stimulated to actively search for novel agents with an improved profile. Despite these caveats, the very existence of these ligands has been revolutionary. They provide an indispensable tool for evaluating therapeutic candidates, by enabling researchers to directly demonstrate a drug’s ability to inhibit tau protein aggregation, prevent the formation of toxic oligomers, and facilitate clearance, thereby opening up new avenues for the treatment of tauopathies.

## 5. Conclusions

AD is a complex, age-related neurodegenerative disorder primarily characterized by the pathological accumulation of Aβ plaques and hyperphosphorylated tau protein. Despite significant research investment, the intricate interplay between diverse pathogenic mechanisms—including neuroinflammation, oxidative stress, cholinergic deficits, and others—remains incompletely elucidated. Accumulating evidence underscores the multifactorial nature of AD, where these interconnected pathways collectively drive disease progression. This complexity highlights the critical need for multi-target therapeutic strategies.

Molecular imaging, particularly positron emission tomography (PET), has revolutionized AD management. Approved amyloid-specific radiotracers (e.g., [^18^F]Flutemetamol, [^18^F]Florbetapir, [^18^F]Florbetaben) and emerging tau-PET ligands (e.g., [^18^F]Flortaucipir) provide in vivo detection of core pathologies, significantly enhancing diagnostic accuracy. Crucially, beyond diagnosis, these imaging biomarkers serve as indispensable tools for therapeutic development. They enable objective assessment of target engagement (e.g., reduction in Aβ/tau burden) and disease progression, facilitating the evaluation of novel therapeutics targeting mechanisms such as Aβ clearance, tau aggregation inhibition, and neuroprotection.

Looking forward, the future of AD treatment lies in seamlessly integrating these advanced diagnostic tools with multi-target therapeutic paradigms. The refinement of molecular imaging techniques is paramount to this mission. Several key directions emerge:

1. Theragnostic integration: Future research must focus on developing parallel imaging and therapeutic ligands targeting the same pathway (e.g., a tau-PET ligands and a tau-aggregation inhibitor). This would allow for direct, real-time monitoring of a drug’s distribution and efficacy at its target site.
2. Combinatorial biomarker tracking: The true potential of multi-target therapies can only be realized with the ability to simultaneously track multiple pathological processes in a patient. This necessitates the development of novel radiotracers for emerging targets like neuroinflammation (e.g., TSPO), synaptic density, and specific immune responses, enabling a holistic view of treatment effects.
3. Personalized treatment algorithms: The ultimate clinical application of this synergy is the creation of personalized treatment algorithms. A patient’s initial PET profile (e.g., high Aβ, low tau; high neuroinflammation) could dictate the choice of combination therapy (e.g., anti-amyloid + anti-inflammatory). Subsequent scans would then objectively guide treatment adjustments, moving away from a one-size-fits-all approach to truly personalized medicine.
4. Advanced Quantification and Artificial Intelligence: Overcoming current limitations in PET quantification (e.g., off-target binding, partial volume effect) through advanced modeling and artificial intelligence is critical. This will enhance the sensitivity to detect subtle treatment-induced changes, essential for proving the efficacy of multi-target drugs that may offer modest but clinically meaningful benefits at each targeted pathway.

Therefore, the advancement of effective multi-target therapies for AD is fundamentally dependent on the continued refinement of molecular imaging techniques. The co-evolution of highly specific PET biomarkers and multi-functional drugs will be the cornerstone of next-generation AD management, transforming our approach from merely diagnosing pathology to dynamically guiding and optimizing complex treatment regimens. This synergistic integration of diagnostics and therapeutics will be essential for overcoming the multifactorial complexity of AD and delivering on the promise of personalized medicine.

## Figures and Tables

**Table 1:** Comparative Analysis of Pathogenic Hypotheses in AD.

Hypothesis	Therapeutic Promise	Challenges and Limitations	Clinical Validation Status	**Diagnostic** **Utility**
**Cholinergic**	- rapid symptomatic effect; - well-known and studied target; - several approved drugs;	- does not modify disease course, only symptoms; - effect is temporary and moderate; - side effects;	**Very High.** AChE inhibitors (donepezil, rivastigmine) are the standard of symptomatic care for decades	**Low;** Not used for diagnosis.
**Amyloid (Aβ)**	- directly targets a key pathological substrate; - multiple therapeutic approaches (antibodies, BACE inhibitors);	- weak correlation with cognitive decline at late stages; - serious side effects (ARIA); - modest clinical effect;	**High.** Several drugs approved (aducanumab, lecanemab, donanemab); effect proven but modest;	**Very High;** Aβ PET imaging and CSF/blood Aβ_42/40_ ratio is standard diagnostic criteria.
**Tau pathology**	- strongest correlation with cognitive decline and brain atrophy; - more “downstream” process closer to neurodegeneration;	- intracellular pathology makes drug access difficult; - risk of disrupting tau’s normal functions;	**Medium/Growing.** Many anti-tau antibodies and other approaches in early/mid-stage clinical trials; no approved drugs yet;	**Very High.** Tau PET imaging and plasma p-tau (p-tau181, p-tau217) are revolutionary biomarkers for diagnosis and staging.
**Neuroinflammation**	- early event in pathogenesis; - validated by human genetics (TREM2, CD33); - potential to boost brain’s “cleanup” system;	- dual role of microglia (protective/destructive); - risk of suppressing necessary immune function; - lack of target specificity;	**Low/Medium.** Therapies (CD33 antagonists, TREM2 agonists) mostly in early (phase I/II) trials; data is preliminary;	**Medium.** PET ligands for activated microglia exist but are less specific and not routine.
**Oxidative stress**	- a common node for many pathological processes; - wide range of potential antioxidants;	- process is highly non-specific; - most clinical trials of antioxidants failed; - challenge of brain delivery;	**Low.** Failures in major clinical trials; new formulations and approaches are in very early stages.	**Low;** Not used for diagnosis.
**Metal ion** **hypothesis**	- unique approach targeting both Aβ aggregation and oxidative stress; - potential for drug repurposing (chelators);	- risk of systemic side effects from disrupting metal homeostasis; - poor BBB permeability for chelators;	**Low.** A few small trials of chelators showed mixed results; no approved therapies.	**Low;** Not used for diagnosis.
**Glutamate** **excitotoxicity**	- explains rapid neuronal loss; - approved drug (memantine) with moderate efficacy;	- memantine provides only symptomatic relief and is for moderate-to-severe stages; - difficulty targeting without disrupting physiological signaling;	**Medium.** Memantine is approved for moderate-to-severe AD; newer receptor-targeting drugs have not succeeded.	**Low;** Not used for diagnosis.
**Microbiota–gut–brain axis**	- non-invasive intervention via probiotics/prebiotics/diet; - potential for very early prevention;	- extremely difficult to establish causality in humans; - mechanisms of interaction a poorly understood; - high individual variability of microbiome;	**Very Low.** Mostly preclinical data; clinical trials of probiotics show mixed results.	**Very Low;** Not used for diagnosis.
**Abnormal** **autophagy**	- fundamental process: targeting it could clear all types of toxic aggregates (Aβ, p-tau); - very high potential;	- extreme difficulty in targeting brain autophagy specifically without severe systemic side effects; - lack of specific and safe activators;	**Low.** Preclinical stages. Existing drugs (Rapamycin) are unsuitable due to side effects; search for safe activators is ongoing;	**Low;** Not used for diagnosis.
**Cellular** **senescence**	- novel and promising direction; - potential to use senolytics (Dasatinib + Quercetin) to clear senescent cells;	- no direct proof of causal role in human AD; - risk of off-target effects and unpredictable long-term consequences of senolytic therapy;	**Very Low.** First pilot studies in humans just beginning; for AD, only preclinical data exists;	**Low;** Not used for diagnosis.
**Endoplasmic** **reticulum stress**	- a key player in proteostasis failure; - potential for drug repurposing (e.g., TUDCA);	- similar challenge as autophagy: hard to target without disrupting vital UPR functions in other organs;	**Low.** Preclinical stages. Some studies of TUDCA in other neurodegenerative diseases (e.g., ALS), but not in AD;	**Low;** Not used for diagnosis.
**Ubiquitin-** **proteasome** **system**	- fundamental cellular “quality control” system; - high specificity of potential targets (individual E3 ligases, DUBs);	- high difficulty in developing drugs for specific E3s/DUBs; - risk of globally disrupting UPS with fatal cellular consequences;	**Very Low.** Purely fundamental and preclinical research; no approaches near clinical trials;	**Low;** Not used for diagnosis.

**Table 2:** Classes of therapeutic agents for the treatment of AD, their molecular targets and current development status. Status abbreviations: FDA—approved by the Office for Sanitary Supervision of the Quality of Food and Medicines of the United States.

Class of Agent	Examples	Mechanism of Action/Target	Status	Key Limitations
**Small Molecules**	Methylene Blue (MB), LMTM	reduces tau protein aggregation	clinical trials	mixed efficacy in trials; delivery issues persist.
	Nitrocatechol derivatives, 5-nitro-α-cyanocarbonamide derivatives	modulate tau protein aggregation	preclinical studies	good anti-aggregation activity in preclinical models (caffeic acid derivatives)
	NQ-DA (naphthoquinone-dopamine hybrid)	targets PHF motifs; inhibits tau aggregation	preclinical studies	effective tau aggregation inhibitor
	LDN193594	inhibits kinases CDK5 and GSK3β	preclinical studies (in vivo)	reduced tau pathology, improved cognition (rodent models).
	SCR1693 (tacrine-based)	promotes tau dephosphorylation; reduces Aβ production	preclinical studies	dual action: reduces Aβ and promotes tau dephosphorylation.
	(R)-[^11^C]PK11195	binds TSPO for neuroinflammation imaging	approved (diagnostic)	FDA-approved for neuroinflammation PET imaging
	D-APV (D-AP5)	NMDA receptor blocker	preclinical studies (ex vivo)	blocks Aβ uptake and neuroinflammation (ex vivo)
	CAPE, TGC86	antioxidant; modulates Aβ aggregation	preclinical studies	efficacy in mice; suppresses amyloid and mitochondrial damage
	PF-04447943	PDE9 inhibitor; increases cGMP signaling	clinical trials	enhances synaptic plasticity and improves memory in preclinical models; prevents dendritic spine loss in Tg2576 mice
	Gliflozins	SGLT2 inhibitor; improves cerebral metabolism, reduces neuroinflammation	clinical trials (repurposing/ phase II)	neuroprotective effects; associated with lower dementia risk and slower cognitive decline in clinical studies
	LDN-57444	inhibitor of ubiquitin-specific protease USP1	preclinical studies	investigated in oncology; modulates response to DNA damage; neuroprotective potential in AD requires separate study
	HBX 41,108	inhibitor of ubiquitin-specific protease USP7	preclinical studies	modulates the stability of key proteins; efficacy and safety in AD have not been studied
	Navitoclax (ABT-263)	Bcl-2/BCL-XL inhibitor (BH3 mimetic)	clinical trials (phase II)	first-generation senolytic; shows efficacy but causes thrombocytopenia
	ABT-737	Bcl-2/BCL-XL inhibitor (BH3 mimetic)	preclinical studies	prototypical BH3 mimetic; research tool for studying senescence
	A-1331852	selective BCL-XL inhibitor	preclinical studies	more selective for BCL-XL; potentially better safety profile
	A-1155463	selective BCL-XL inhibitor	preclinical studies	highly selective BCL-XL inhibitor; reduces senescent cell burden
	Dasatinib + Quercetin	senolytic combination (kinase inhibition + flavonoid)	clinical trials (phase I)	reduces senescent cell burden in patients with diabetic kidney disease
	17-DMAG (Alvespimycin)	HSP90 inhibitor	preclinical studies	reduces senescent cell load in animal models of aging and disease
	PIK3R3 inhibitors	p53/p21 signaling pathway inhibitor	preclinical studies	emerging target for s elective senolysis
	TRIAP1 inhibitors	p53/p21 signaling pathway inhibitor	preclinical studies	novel approach to target senescent cells
	Bocodepsin (OKI-179)	HDAC inhibitor	preclinical studies	epigenetic modulator of senescence; shows potential in cancer models
	SB203580	p38MAPK inhibitor	preclinical studies	reduces SASP production and senescent cell viability
	UR13756	p38MAPK inhibitor	preclinical studies	attenuates senescence- associated inflammation
	BIRB796	p38MAPK inhibitor	preclinical studies	potent p38 inhibitor with senomorphic activity
	AG490	JAK/STAT inhibitor	preclinical studies	reduces inflammatory SASP components
	Momelotinib	JAK/STAT inhibitor	preclinical studies	suppresses senescence-associated inflammation
	INCB18424	JAK/STAT inhibitor	preclinical studies	attenuates SASP and chronic inflammation
	GW2580	CSF1R inhibitor (modulates microglia)	preclinical studies	alleviated Aβ accumulation as well as neuritic and synaptic damage by targeting microglia
	Genetic clearance (p16-3MR model)	Inducible elimination of p16+ senescent cells	preclinical studies	treatment with AP20187 improved cognitive function, demonstrating proof-of-concept for whole-body senescent cell clearance
	Rapamycin	mTOR inhibitor	clinical trials (phase II)	extends health span, reduces SASP, improves function in aged models
	Rapalogs (e.g., Everolimus)	mTOR inhibitor	clinical trials	show potential in targeting age-related pathologies
	Torin 1	mTOR inhibitor	preclinical studies	second-generation mTOR inhibitor; potent senomorphic effects
	NVP-BEZ235	PI3K/mTOR inhibitor	preclinical studies	dual inhibitor with potential senomorphic activity
	KU-60019	ATM kinase inhibitor	preclinical studies	modulates DNA damage response to suppress senescence
	KU-55933	ATM kinase inhibitor	preclinical studies	attenuates senescence phenotypes
	Loperamide	Ca^2+^ channel inhibitor	preclinical studies	modulates calcium signaling to disrupt SASP
	NDGA	Ca^2+^ channel inhibitor	preclinical studies	shows senomorphic activity in various models
	Isradipine	Ca^2+^ channel inhibitor	preclinical studies	potential senomorphic effects through calcium modulation
	Simvastatin	ERK pathway inhibitor	preclinical studies	pleiotropic effects including potential senomorphic activity
	CDD-111	MAPK inhibitor	preclinical studies	reduces senescence-associated phenotypes
	Anakinra	IL-1 receptor antagonist	clinical trials (for other indications)	reduces inflammation; potential senomorphic effects
	Ruxolitinib	JAK1/2 inhibitor	approved (for myelofibrosis)	being repurposed for senescence-related inflammation
	Metformin	AMPK activator; NF-κB inhibition	approved (for type 2 diabetes)	preclinical and epidemiological data suggest potential benefits for brain health
	Aspirin	modulates SIRT1; reduces DNA damage response	repurposed/ investigational	associated with reduced risk of some age-related diseases.
**Peptides**	RVG29	targets nAChR for delivery (e.g., BACE1 siRNA)	preclinical studies (in vivo)	55% Aβ reduction in mice; stability challenges
	GHK (glycyl-l-histidyl-l-lysine)	antioxidant, improves TGFβ1 signaling	preclinical studies	endogenous antioxidant; improved cognition (aging mice)
	GSH (Glutathione)	antioxidant protection	preclinical studies	levels reduced in AD; raising them is a therapeutic goal
	NAC (N-acetyl-l-cysteine)	increases GSH levels, antioxidant	preclinical studies	boosts antioxidant enzymes (preclinical)
	D-TLKIVW	inhibits tau aggregation (D-amino acid peptide)	preclinical studies	D-amino acid design enhances stability
	Ornithine-linked peptidomimetics	inhibits aggregation of PHF motifs	preclinical studies	inhibits PHF aggregation via β-helical conformation
	KLVVF, P4, P5 peptides	inhibits tau aggregation	preclinical studies	prevents tau toxicity by retaining random coil state
	ALAPYIP (VHL peptide)	induces ubiquitin-dependent tau degradation	preclinical studies	reduces tau levels in primary neurons and transgenic mouse models
	iAβ5	inhibits aggregation of Aβ and tau	preclinical studies	inhibits both Aβ and tau aggregation (in vitro/in vivo)
	Hairpin peptide mimetics	inhibits Aβ aggregation	preclinical studies	anti-Aβ aggregation via piperidine-pyrrolidine moieties
**Antibodies**	Bapineuzumab	targets N-terminals of Aβ42	clinical trials (phase III completed)	limited efficacy, ARIA side effects
	Solanezumab	targets Aβ13-28 segment	clinical trials (phase III)	safe, reduces Aβ, but no cognitive benefit
	Gantenerumab	targets N-terminal and central regions of Aβ	clinical trials (phase III)	mixed efficacy and safety results
	Crenezumab	binds Aβ oligomers, fibrils, and plaques	clinical trials (phase II/III)	inhibits and disrupts Aβ aggregates
	Aducanumab	targets conformational epitope of Aβ fibrils	approved (FDA, conditional)	conditionally approved; efficacy remains controversial
	DC8E8	identifies tau epitope (residues 294–305)	preclinical studies	basis for the AADvac1 vaccine
	AADvac1 (peptide vaccine)	derived from tau residues 294–305	clinical trials (phase II completed)	safe but no cognitive improvement
	C2N-8E12 (ABBV-8E12)	targets tau protein	clinical trials (phase I completed)	reduced tau, improved cognition; good safety
	Gouranemab (BIIB092)	targets N-terminal fragment of tau	clinical trials (phase I completed)	well-tolerated; development ongoing
	Ta1505	targets pSer413 of tau	preclinical studies (in vivo)	reduces tau, improves synapses and cognition (mice)
	43D, 77 × 10^9^	target tau epitopes 6–18 and 184–195	preclinical studies	significant tau reduction and cognitive restoration
	Tocilizumab	IL-6 inhibitor	approved (for autoimmune diseases)	being explored for neuroinflammation and senescence
	Siltuximab	IL-6 inhibitor	approved (for other indications)	potential senomorphic activity through IL-6 neutralization
	Sirukumab	IL-6 inhibitor	clinical trials	investigated for inflammatory conditions
	Adalimumab	TNF-α inhibitor	approved (for autoimmune diseases)	potential senomorphic effects through TNF-α inhibition
	Etanercept	TNF-α inhibitor	approved (for autoimmune diseases)	shows promise in reducing inflammation
	Infliximab	TNF-α inhibitor	approved (for autoimmune diseases)	potential application in senescence-associated inflammation
**Natural Ligands**	Vitamin E, Selenium	antioxidants	clinical studies	minimal improvement in patients; mixed results
	Flavonols	antioxidants	epidemiological study	epidemiological link to reduced AD risk
	Luteolin	antioxidant, anti-inflammatory, inhibits Aβ and tau aggregation	preclinical studies	multifunctional flavonol; shows promise in reducing key pathologies
	Resveratrol	antioxidant, anti-inflammatory	clinical trials (phase II)	mixed results in patients
	Fisetin	antioxidant, anti-inflammatory	preclinical studies (in vivo)	neuroprotective; reduces oxidative stress (aging rats)
	Tauroursodeoxycholic acid (TUDCA)	anti-apoptotic, reduces oxidative stress, improves mitochondrial function, promotes Aβ clearance	clinical trial	neuroprotective bile acid; currently in trials for AD
	Acitretin	activates α-secretase	clinical study (pilot)	increases sAPPα, decreases Aβ
	Sodium oligomannate	inhibits Aβ aggregation, modulates gut microbiota	approved (China)	modulates microbiota
	Coconut oil	source of ketone bodies	clinical trial (pilot)	improved cognition with diet
	Curcumin derivative PE859	targets both Aβ and tau aggregation	preclinical studies (in vivo)	reduces Aβ/tau, improves cognition (mice)
	EGb 761 (Ginkgo extract)	AChE inhibition, antioxidant	clinical studies	cognitive improvement debated
	Ginkgolide A	attenuates Aβ-induced depolarization, inhibits NMDA receptors	preclinical studies (in vivo)	attenuates Aβ toxicity, inhibits NMDA-R
	Nitidine, Avicin	dual AChE and BuChE inhibition	in vitro studies	dual AChE/BuChE inhibition and anti-aggregation
	Helminthosporin	Dual AChE and BuChE inhibition	in vitro studies	dual AChE/BuChE inhibition, high BBB permeability
	Chrysophanol	AChE and BACE1 inhibition, anti-neuroinflammatory	preclinical studies	anthraquinone with multi-target potential
**Hybrid Molecules**	APH-1105	α-secretase activator (nanoparticle)	clinical trials (phase II ongoing)	intranasal delivery
	ML	controls Aβ aggregation, metal chelation, antioxidant	preclinical studies	reduces Aβ-metal toxicity, decreases ROS, BBB permeable
	DMPD	controls Aβ aggregation, metal chelation	preclinical studies (in vivo)	reduced Aβ, reversed memory loss (AD mice)
	Ber-D	enhanced metal chelation and antioxidant properties	in vitro studies	antioxidant; protects neuronal cells
	Distyrylbenzene-based theranostic	metal chelation, antioxidant, reduces Aβ/tau	preclinical studies	theranostic potential: imaging and therapy

**Table 3:** Comparative overview of Aβ-specific PET radiopharmaceuticals. Key advantages, disadvantages and clinical application status are given. The main compromise is between the affinity of Aβ, pharmacokinetics (penetration through BBB and clearance) and the practical applicability determined by the half-life of the isotope. Status abbreviations: FDA—approved by the Office for Sanitary Supervision of the Quality of Food and Medicines of the United States; EMA—by the European Agency of Medicines.

Radiopharmaceuticals	Isotope	Advantages	Key Limitations	Status
ThT	-	historical marker of Aβ fibrils	low specificity, does not penetrate the BBB	preclinical studies
6-Me-BTA-1	-	high affinity for Aβ_42_	not applicable in vivo use	preclinical studies
[^11^C]6-Me-BTA-1	^11^C	prototype for PiB	short t½ (20 min)	preclinical studies
PiB ([^11^C]PIB)	^11^C	gold standard for Aβ-PET	limited scanning time	preclinical studies
[^18^F]Flutemetamol	^18^F	analogue of PiB with a long t½ (110 min)	high non-specific binding to white matter	approved (FDA, EMA)
[^11^C]SB-13	^11^C	selectivity for dense plaques	low permeability across the BBB	preclinical studies
[^18^F]FMAPO	^18^F	low background in white matter	relatively moderate affinity	preclinical studies
[^18^F]Florbetaben	^18^F	high signal to noise ratio	slow accumulation kinetics (60–90 min)	approved (FDA)
[^18^F]Florbetapir	^18^F	fast accumulation (20 min)	moderate lipophilicity	approved (FDA, EMA)
[^18^F]FIBT	^18^F	ultra-high affinity	complex synthesis, limited clinical data	clinical trials (phase I/II)

**Table 4:** Comparative overview of tau-specific PET radiopharmaceuticals. Key advantages, disadvantages, and clinical application status are presented. The main trade-offs are between affinity for tau pathology, selectivity (lack of cross-linking to Aβ and other targets), pharmacokinetics, and suitability for quantitative analysis. A particular problem for many ligands is non-specific binding to white matter. Status abbreviations: FDA—approved by the of the United States Food and Drug Administration.

Radiopharmaceuticals	Isotope	Advantages	Key Limitations	Status
[^18^F]FDDNP	^18^F	first ligand for in vivo tau imaging	high binding to Aβ, slow kinetics, low signal-to-noise ratio	preclinical studies
BF-126	^18^F/^11^C	fast accumulation (30–40 min), suitable for dynamic studies	moderate cross-reactivity with Aβ	preclinical studies
BF-158/[^11^C]BF-158	^18^F/^11^C	good permeability through the BBB, stable pharmacokinetics	limited clinical data	clinical trials (phase I)
BF-170	^18^F	better selectivity (almost no binding to Aβ), ultra-high affinity	complex synthesis	clinical trials (phase II)
[^18^F]THK5105	^18^F	sensitivity to early stages of tauopathies	slow clearance, white matter artifacts	preclinical studies
[^18^F]THK5117	^18^F	improved version of THK5105 with less non-specific binding	limited availability	preclinical studies
[^11^C]PBB3	^11^C	3R/4R-tau imaging, fast clearance (suitable for repeat scans)	short half-life (^11^C), high cost	clinical trials
Flortaucipir ([^18^F]T807)	^18^F	FDA approved (Tauvid), high specificity for parenchymal tau	slow kinetics (60–80 min before scanning)	approved (FDA)
[^18^F]T808	^18^F	fast accumulation (30 min), specificity comparable to T807, fewer artifacts	limited validation data	preclinical studies

## Data Availability

Not applicable.
